# Sleep traits and risk of end-stage renal disease: a mendelian randomization study

**DOI:** 10.1186/s12920-023-01497-9

**Published:** 2023-04-07

**Authors:** Kaixin Li, Jiaxi Zhao, Wenjing Yang, Zhibin Ye

**Affiliations:** 1 Department of Nephrology, Shanghai Key Laboratory of Clinical Geriatric Medicine, Huadong Hospital Affiliated to Fudan University, Shanghai, 200040 China; 2 General Practice Ward/International Medical Center Ward, General Practice Medical Center, West China Hospital, Sichuan University, Chengdu, 610041 Sichuan China; 3 Huadong Hospital Affiliated to Fudan University, Shanghai, 200040 China

**Keywords:** Mendelian randomization, End-stage renal disease, Sleep traits, Insomnia, Snoring

## Abstract

**Background:**

Epidemiological evidence relating sleep disorders to end-stage renal disease (ESRD) has been obscure. The present study is sought to examine the association between sleep traits and ESRD.

**Methods:**

For this analysis, we selected genetic instruments for sleep traits from published genome-wide association studies (GWAS). As instrumental variables, independent genetic variations linked with seven sleep-related features (sleep duration, getting up in the morning, daytime napping, chronotype of morning/evening person, sleeplessness/insomnia, non-snoring, and daytime dozing) were chosen. A two-sample Mendelian randomization (TSMR) study was conducted to assess the causal relationship between sleep traits and ESRD (N = 33,061). The reverse MR analysis subsequently determined the causal relationship between ESRD and sleep traits. The causal effects were estimated using inverse variance weighted, MR-Egger, weighted median. To conduct sensitivity studies, Cochran’s Q test, MR-Egger intercept test, MR-PRESSO, leave-one-out analysis, and funnel plot were used. To study the potential mediators, multivariable mendelian randomization analyses were undertaken further.

**Results:**

Genetically predicted sleeplessness/ insomnia (OR = 6.11, 95%CI 1.00-37.3, P = 0.049, FDR = 0.105), getting up in the morning easily(OR = 0.23, 95%CI 0.063–0.85; P = 0.0278, FDR = 0.105), non-snoring (OR = 4.76E-02, 95%CI 2.29E-03-0.985, P = 0.0488, FDR = 0.105) was suggestively associated with the risk of ESRD. However, we found no evidence favoring a causal association between other sleep traits and ESRD through the IVW method.

**Conclusion:**

The present TSMR found no strong evidence of a bidirectional causal association between genetically predicted sleep traits and ESRD.

**Supplementary Information:**

The online version contains supplementary material available at 10.1186/s12920-023-01497-9.

## Introduction

Chronic kidney disease (CKD) is an international health issue [1]. A considerable number of CKD patients will progress to end-stage renal disease (ESRD) [2]. Therefore, finding preventative strategies for ESRD is especially important. Previous epidemiological studies have pointed out some potential risk factors to increase the incidence of ESRD. Significant roles are played by diabetes, hypertension, and obesity in the development of ESRD [3]. However, the etiology of ESRD remains obscure. Exploring the pathophysiology of ESRD aids in developing preventative and therapeutic measures.

The prevalence of sleep problems in patients with CKD has been shown to be significant, with roughly 50% of patients suffering from poor sleep quality or insomnia [4], and 44% of patients with ESRD experiencing sleep disturbance [5]. Several studies indicated that sleep was one of the potential risk factors for the development and progression of CKD [6, 7]. A prospective cohort study found that individuals with poor sleep habits had an elevated risk of CKD [7]. In addition, sleep disorder is frequently linked to obesity [8, 9], metabolic syndrome [10], diabetes [11], and hypertension [12], all of which hasten the progression of renal disease [13]. A cohort study identified poor sleep quality as a predictor of ESRD and found that both short (5 h) and long (8 h) sleep duration were linked to the risk of ESRD [6]. However, researchers were unable to determine if sleep disruption raises the risk of ESRD.

Mendelian randomization (MR) analysis uses genetic variants as instrumental variables (IVs) for exposure, which reduces measurement error and bias. MR is used to test exposure-outcome causal inferences. Two-sample MR (TSMR) allows IV analysis when the exposure and the outcome variables are measured in two independent datasets, so this approach can be particularly valuable when applied to large datasets that exist relating GWAS data to disease outcomes, but which lack intermediate phenotype data [14]. We conducted a TSMR research to examine the causative influence of seven sleep traits on ESRD.

## Methods

### Study design

**Fig. 1:**
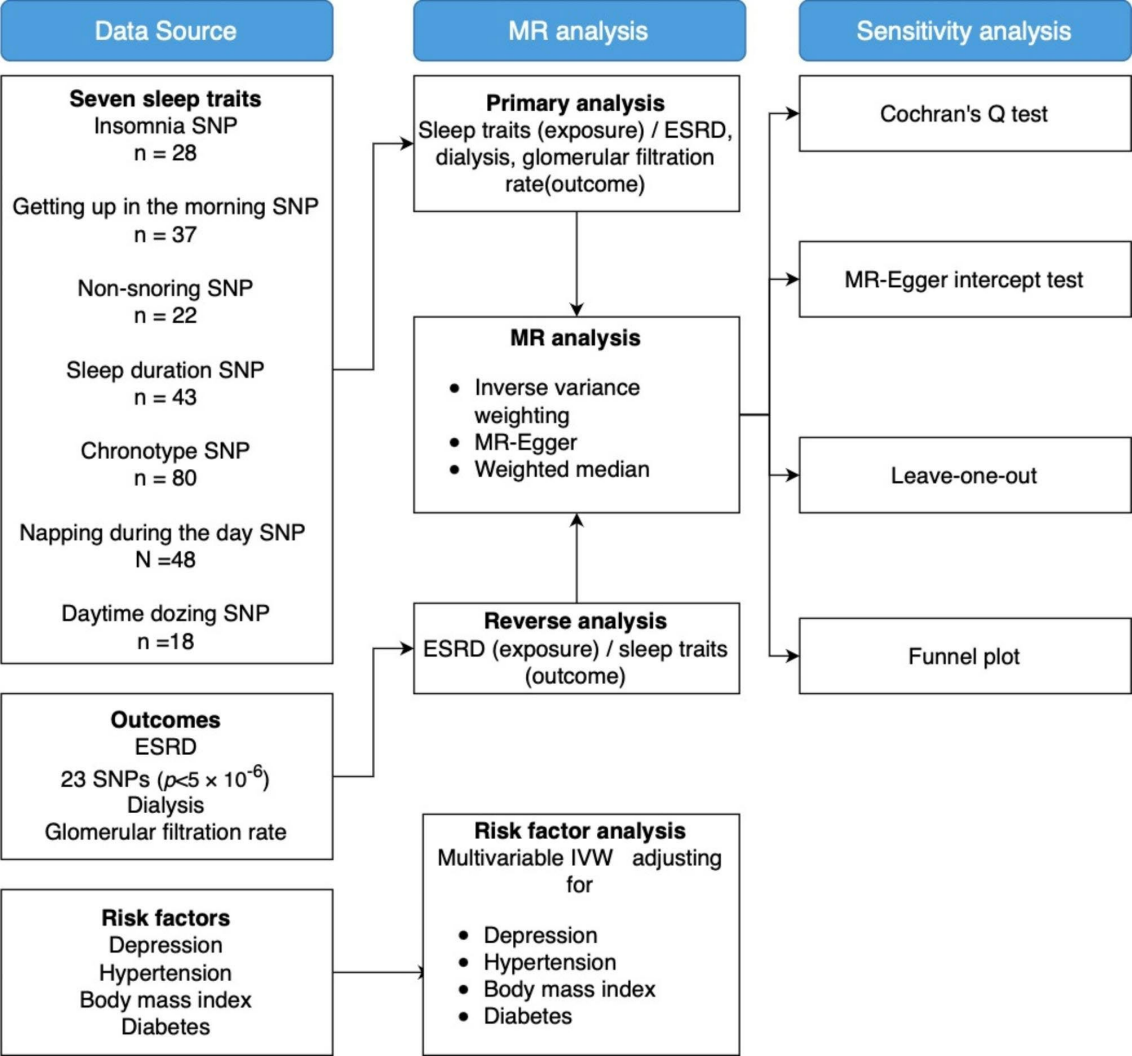
Overview of study design. BMI indicates body mass index; ESRD, End-stage renal disease; GWAS genome-wide associations study; IVW inverse-variance weighted; SNP single-nucleotide polymorphisms. Data extracted were beta coefficients with corresponding standard errors of the SNP-insomnia, SNP-getting up in the morning, SNP-non-snoring, SNP-sleep duration, SNP- morning chronotype, SNP-napping during the day, SNP-daytime dozing, SNP-ESRD, SNP-dialysis, SNP-glomerular filtration rate, SNP-depression, SNP-BMI, SNP-hypertension and SNP-diabetes associations

Figure 1 shows the overview of the study design. We conducted a bidirectional MR analysis to assess sleep traits’ association with ESRD. Setting dialysis and glomerular filtration rate as additional outcomes to supplement results, and then we conducted multivariable MR to test the true causal association between sleep traits and ESRD. All statistical analyses were performed using the two-sample MR package (version 0.5.6) [15] and MR-PRESSO package (version 1.0) [16] in R (version 4.2.1). The study methods were compliant with the STROBE-MR checklist [17] (Supplementary Tables 12, Additional File 1).

### Data sources

Sleep-related characteristics and outcome data sources have been gathered and made accessible online, an additional file shows this in more detail (see Supplementary Tables 1, Additional File 1). Since this study was based on published data, no ethical approval nor informed consent was required.

### Exposures

Summary statistics from IEU open GWAS project were used as the data for sleep traits [18], which included 337000 unrelated individuals aged from 40 to 69 years old from a study of UK Biobank between 2006–2010 [19]. Instrumental Variables (IVs) were chosen from previously known single-nucleotide polymorphisms (SNPs) related to each sleep trait (exposure). Insomnia was characterized as difficulty falling asleep at night or waking up in the middle of the night. Getting up in the morning was evaluated based on how easy it was to locate, and the significant self-report was fairly easy. The genetic connection to non-snoring was based on snoring complaints from a partner, relative, or friend. The genetic relationship of sleep duration was determined by asking people how many hours they slept per day, and the units of measurement were hours per day. Chronotype is the natural propensity for the individual to sleep at a particular time, and the morning chronotype is someone who self-reported as more of a ‘morning’ person than an ‘evening’ person.

### Outcome

Summary statistics for ESRD were downloaded from the NHGRI-EBI GWAS Catalog (Buniello, MacArthur, et al., 2019) for study GCST008031 (Wojcik GL et al. 2019) downloaded on 01/09/2022. ESRD was defined as an eGFR (by the CKD-Epi Equation) of < = 15 ml/min/1.73m^2^. ESRD was modeled as a binary outcome, and models were adjusted for age, sex, race/ethnicity, study, and study center, etc. The study comprised 33,061 individuals, including 602 cases and 32,459 controls [20]. The summary statistics for glomerular filtration rate (GFR) were downloaded from the NHGRI-EBI GWAS Catalog (Buniello, MacArthur, et al., 2019) on 17/12/2022 for study (GCST003375) including 32,834 European ancestry individuals. The assessment of GFR was based on cystatin C, and GFRcys was estimated as 76.7 × (serum cystatin C)^−1.19^ [21]. The outcome summary statistics for dialysis were obtained from the 5th release of the FinnGen study with 648 cases and 212,841 controls [22].

### Risk factors

Summary statistics for diabetes were downloaded from the NHGRI-EBI GWAS Catalog (Buniello, MacArthur, et al., 2019) for study GCST006867 downloaded on 17/12/2022 [23]. The summary statistics for depression and hypertension were obtained from the 5th release of the FinnGen study [22]. Genetic instrumental variables for BMI were obtained from the Genetic Investigation of Anthropometric Traits (GIANT) Consortium via the IEU Open GWAS [15].

### Selection of instrumental variables

To determine the optimal IVs for sleep, we first extracted SNPs from published data strongly associated with sleep traits (*p* < 5 × 10^− 8^). Linkage disequilibrium (LD) SNPs were eliminated (r2 0.001, clumping window = 10,000 kb) to ensure exposure instrument independence. Then, we extracted the sleep trait instrumental factors from the ESRD GWAS and eliminated palindromic SNPs. After harmonizing exposure and outcome data, we discovered that the same allele affects both exposure and outcome. Last, we filtered SNPs with *F*-statistics greater than 10 to ensure instrument reliability and eliminate bias to satisfy the relevance assumption of MR analysis that genetic instruments were associated with the risk factor of interest. 28 SNPs for sleeplessness/insomnia, 37 for getting up in the morning, 22 for non-snoring, 44 for sleep duration, 80 for morning chronotype, 48 for napping during the day, and 18 for daytime dozing were extracted for MR analysis. The total *F* value for sleep duration, getting up in the morning, morning chronotype, napping during the day, insomnia, non-snoring, and daytime dozing were 660.15, 547.39, 1321.85, 935.98, 423.11, 334.99, and 315.36, respectively, and the phenotypic variation explained (PVE) for each exposure were 0.0019, 0.0016, 0.004, 0.0028, 0.0013, 0.0011, and 0.001, respectively. In the reverse MR analysis, a more relaxed threshold was used (*p* < 5 × 10^− 6^) to select more SNPs of ESRD, which had been previously used in MR research [24]. We extracted 23 SNP for ESRD with a total *F*-statistic of 124.05 and then removed the SNPs with *F* < 10 to satisfy the first MR assumption. Finally, we integrated 7 SNPs for ESRD. The PVE for ESRD was 0.23. The additional file (see Supplementary Tables 2–9, Additional File 1) provided an overview of GWAS datasets and related SNPs.

### Mendelian randomization analysis

Using random-effects Inverse-variance weighted (IVW), MR Egger, and Weighted median, we examined if sleep traits caused ESRD risk. We chose IVW as the primary technique for MR analysis. In addition, false discovery rate (FDR) adjusted p-values proposed by Benjamini and Hochberg were used to address multiple correction testing [25]. An FDR lower than 0.05 indicated statistical significance and supported strong evidence of a causal relationship. Associations with *p* < 0.05 but FDR > 0.05 were regarded as suggestive evidence of association.

### Sensitivity analysis

Cochran’s Q test, the MR-Egger regression test and the Mendelian Randomization Pleiotropy Residual Sum and Outlier (MR-PRESSO) test were used to identify heterogeneity or pleiotropy [16, 26, 27]. To check reproducibility, we ran a sensitivity analysis utilizing the leave-one-out technique.

### Multivariable MR

To determine whether the genetic instruments were associated with the risk factor of interest, were independent of potential confounders, and could only affect the outcome through the risk factor and not through alternative pathways, we conducted multivariable MR using genetic variants associated with numerous, potentially connected exposures to estimate the effect of each exposure on a single outcome [28]. We included some risk factors as potential confounders in the sleep traits and ESRD relationship. The multivariable MR was applied to test whether there was a true causality between sleep traits and ESRD. Only causality suggested by the primary MR analysis would undergo further tests.

## Results

### Primary MR analysis for the association between sleep traits and ESRD

**Fig. 2:**
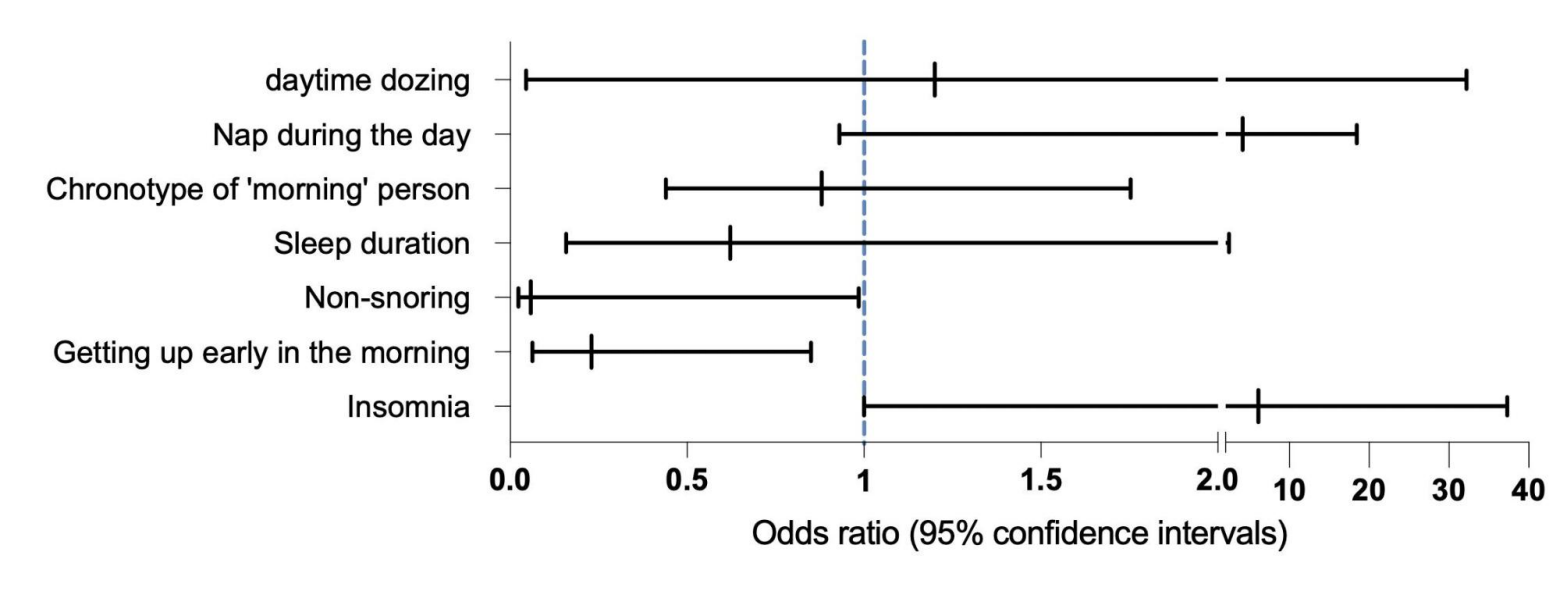
Associations of genetic liability to 7 sleep traits with ESRD. Genetic liability to Non-snoring, getting up early, and insomnia was suggestively associated with ESRD in the primary analysis. Estimates are from the random-effects inverse variance weighted method

**Fig. 3:**
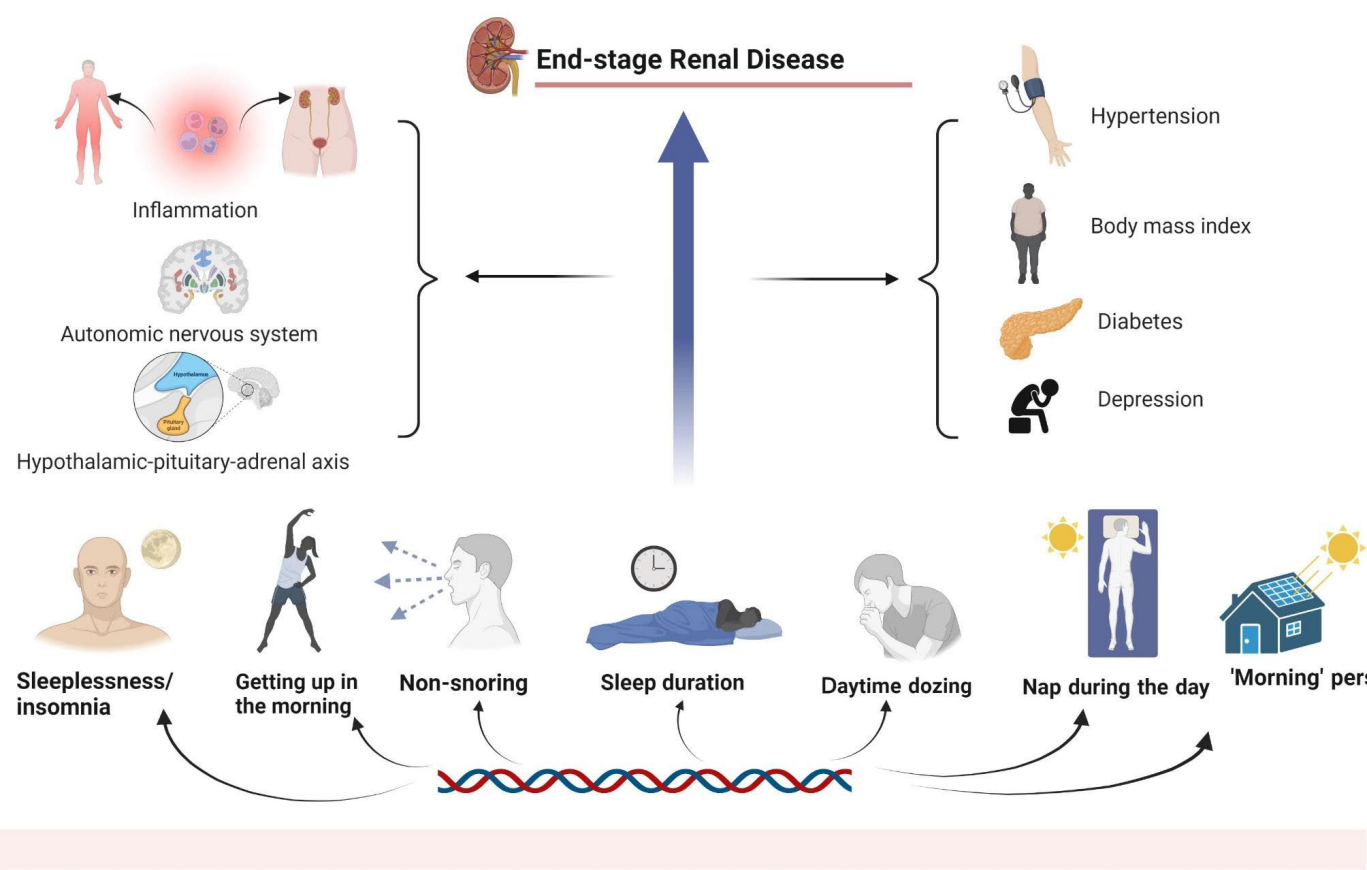
Genetic liability to sleep traits in relation to end-stage renal disease

**Fig. 4:**
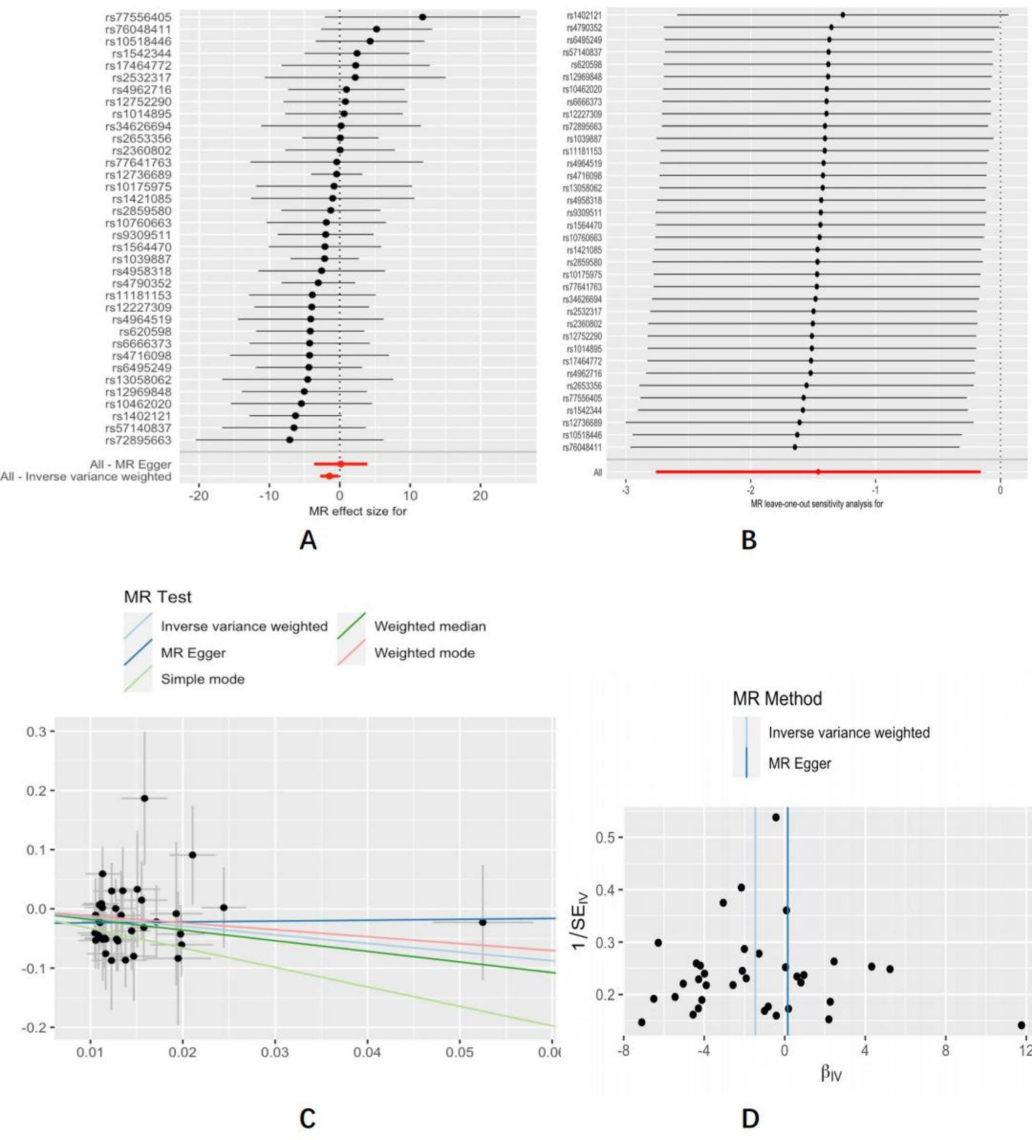
Forest plot **(A)**, leave-one-out sensitivity analysis **(B)**, scatter plot **(C)**, and funnel plot **(D)** of the suggestive causal effect of getting up in the morning on ESRD risk

According to IVW analysis, there were suggestive associations between sleeplessness/ insomnia (OR = 6.11, 95%CI 1.00-37.3, P = 0.049, FDR = 0.105, Power = 0.95), Getting up easily in the morning (OR = 0.23, 95%CI 0.063–0.85; P = 0.0278, FDR = 0.105, Power = 0.1)(Fig. 4), non-snoring (OR 4.759E-02, 95%CI 2.29E-02–0.985, P = 0.0488, FDR = 0.105, Power = 0.12) and the risk of ESRD (Table 1). For other sleep behaviors, we found no evidence of associations between genetically-predicted sleep duration (OR = 0.622, 95%CI 0.158–2.447, P = 0.49, FDR = 0.686) (See Supplementary Fig. 1, Additional File 1), the morning chronotype (OR = 0.88, 95%CI 0.44–1.753, P = 0.73, FDR = 0.851) (See Supplementary Fig. 2, Additional File 1) and ESRD. Similarly, daytime dozing (0R = 1.2, 95%CI 4.48E-02–3.21E + 02, P = 0.91, FDR = 0.91) (See Supplementary Fig. 6, Additional File 1)and rarely daytime napping (OR = 4.15, 95%CI 0.93–18.44, P = 0.06, FDR = 0.105) (See Supplementary Fig. 3, Additional File 1) was not observed evidence of having a causal association with ESRD risk. (Figures 2 and 3).

**Table 1:** The causal relationship between sleep traits and ESRD, dialysis and glomerualr filtration rate

Exposure	Outcome	IVW				MR Egger			Weighted median		
		OR/b	95%CI	P	FDR	OR/b	95CI	P	OR/b	95CI	P
Sleeplessness/insomnia	ESRD	6.117	1.004-3.72e + 01	0.049	0.105	1038.81	0.007-1.63e + 08	0.26	4	0.324-4.92e + 01	0.28
Sleep duration		0.622	0.158–2.446	0.49	0.686	0.131	0.0005–37.97	0.48	1.81	0.275–11.917	0.53
Morning chronotype		0.889	0.447–1.767	0.73	0.8517	2.68	0.486–14.754	0.26	1.296	0.454–3.698	0.68
Getting up in the morning		0.232	0.063–0.853	0.028	0.105	1.16	0.027–50.53	0.938	0.166	0.027–1.015	0.051
Non-snoring		4.76E-02	2.3e-03-0.985	0.049	0.105	1.10E-06	2.22e-14-54.17	0.145	3.75E-02	5.03e-04-2.79	0.134
Daytime dozing		1.2	4.48d-02-3.21e + 01	0.91	0.91	831.7	1.94e-07-3.56e + 12	0.56	5.75	3.46e-02-3.14e + 02	0.607
Nap during the day		4.159	0.938–18.449	0.06	0.105	259.31	0.405-165921.23	0.099	7.06	0.846–58.93	0.07
ESRD	Sleeplessness/insomnia	0.997	0.990–1.003	0.324		1.031	0.975–1.089	0.329	0.998	0.989–1.007	0.720
	Sleep duration	-0.001	-0.008-0.006	0.783		-0.022	-0.08-0.036	0.489	-0.002	-0.01-0.007	0.712
	Morningchronotype	0.994	0.986–1.003	0.173		0.995	0.925–1.005	0.912	0.994	0.984–1.005	0.315
	Getting up in the morning	1.002	0.995–1.008	0.616		0.981	0.926–1.039	0.539	1.001	0.992–1.009	0.862
	Non-snoring	1.002	0.995–1.010	0.543		0.967	0.903–1.036	0.388	1.000	0.994–1.006	0.994
	Daytime dozing	1.001	0.996–1.006	0.598		1.045	1.007–1.085	0.068	0.999	0.993–1.006	0.831
	Nap during the day	0.995	0.989–1.001	0.088		1.012	0.964–1.063	0.648	0.998	0.991–1.005	0.552
Sleeplessness/insomnia	Dialysis	0.485	0.055-4.28e + 00	0.514	0.681	446.026	1.721-1.156e + 05	0.041	0.555	0.032-9.433e + 00	0.684
Sleep duration		0.303	7.00e-02-1.31	0.11	0.681	0.009	2.49e-05-3.42	0.12	0.341	4.09e-02-2.85	0.32
Morning chronotype		0.619	0.279–1.373	0.238	0.681	0.354	0.053–2.374	0.288	0.287	0.092–0.888	0.03
Getting up in the morning		0.538	0.096–3.002	0.877	0.877	0.184	0.0003–99.841	0.601	0.554	0.055–5.534	0.615
Non-snoring		6.901	1.088e-01-4.376e + 02	0.362	0.681	17373.55	2.635e-05-8.304e + 12	0.349	11.224	1.239e-01-1.016e + 03	0.293
Daytime dozing		3.011	9.93e-02-91.304	0.527	0.681	0.0005	3.105e-11-7589.52	0.379	1.465	1.453e-02-147.69	0.871
Nap during the day		1.627	0.285–9.307	0.584	0.681	3.093	0.004-2428.268	0.741	1.558	0.154–15.719	0.707
Sleeplessness/insomnia	Glomerular filtration rate	-0.0007	-0.143-0.142	0.993	0.993	-0.227	-1.382-0.927	0.708	-0.008	-0.172-0.155	0.921
Sleep duration		-0.018	-0.122-0.086	0.730	0.9765	0.123	-0.485-0.731	0.697	-0.058	-0.177-0.061	0.338
Morning chronotype		-0.015	-0.064-0.033	0.542	0.9765	0.089	-0.144-0.322	0.460	-0.002	-0.063-0.059	0.954
Getting up in the morning		0.021	-0.093-0.136	0.715	0.9765	0.064	-0.497-0.624	0.827	0.053	-0.068-0.175	0.387
Non-snoring		0.232	0.017–0.447	0.034	0.119	-0.478	-2.435-1.478	0.643	0.239	0.001–0.478	0.049
Daytime dozing		0.023	-0.197-0.242	0.837	0.9765	-0.209	-1.772-1.353	0.799	-0.072	-0.285-0.142	0.489
Nap during the day		-0.078	-0.149- -0.006	0.033	0.119	-0.055	-0.384-0.275	0.748	-0.077	-0.171-0.018	0.113

### MR analysis for the association between sleep traits and other outcomes

Setting dialysis to the outcome, based on IVW analysis, revealed no evidence of a causal association between sleep duration (OR = 0.303, 95%CI 7.00E-2–1.31, P = 0.11, FDR = 0.681), sleeplessness/insomnia (OR = 0.485 95%CI 0.055-4.28e + 00, P = 0.514, FDR = 0.681), chronotype (OR = 0.619, 95%CI 0.279–1.373, P = 0.238, FDR = 0.681), getting up in the morning (OR = 0.538, 95%CI 0.096–3.002, P = 0.877, FDR = 0.877), non-snoring (OR = 6.901, 95%CI 1.088E-01-4.376E + 02, P = 0.362, FDR = 0.681), daytime dozing (OR = 3.011, 95%CI 9.93E-02-91.304, P = 0.527, FDR = 0.681), nap during the day (OR = 1.627, 95%CI 0.285–9.307, P = 0.584, FDR = 0.681) and dialysis.

In the analysis of the effect of sleep traits on glomerular filtration rate (GFR), there was no evidence of an effect of sleeplessness/ insomnia (b = -0.0007, 95%CI -0.143-0.142, P = 0.993, FDR = 0.993), sleep duration (b = -0.018, 95%CI-0.122-0.086, P = 0.730, FDR = 0.9765), chronotype (b = -0.015, 95%CI -0.064-0.033, P = 0.542, FDR = 0.9765), getting up in the morning (b = 0.021, 95%CI-0.093-0.136, P = 0.715, FDR = 0.9765), daytime dozing (b = 0.023, 95%CI -0.197-0.242, P = 0.837, FDR = 0.9765) on GFR based on the IVW method. There were suggestive evidence of effects of genetic liability to non-snoring (b = 0.232, 95%CI 0.017–0.447, P = 0.034, FDR = 0.119) and napping during the day (b = -0.078, 95%CI -0.149-0.006, P = 0.033, FDR = 0.119) on GFR.

### Sensitivity analysis

In the sensitivity analysis, we conducted funnel pot, Cochran’s Q test, leave-one-out analysis, and MR-Egger intercept tests. The MR-Egger regression test revealed no horizontal pleiotropy for sleeplessness/insomnia (Intercept=-0.06, P = 0.4), sleep duration (Intercept = 0.021, P = 0.58), morning chronotype (Intercept=-0.024, P = 0.17), getting up easily in the morning (Intercept=-0.025, P = 0.38), non-snoring (Intercept = 0.089, P = 0.245), daytime dozing (Intercept= -0.053, P = 0.567), nap during the day (Intercept=-0.046, P = 0.204). The Cochran’s Q test revealed no heterogeneity in the IVW results for insomnia (Q = 21.32, P = 0.67), sleep duration (Q = 45.45, P = 0.256), morning chronotype (Q = 84.24, P = 0.268), getting up easily in the morning (Q = 20.28, P = 0.978), daytime dozing (Q = 12.62, P = 0.761), non-snoring (Q = 13.7, P = 0.85), daytime dozing (Q = 12.62, p = 0.761), nap during the day (Q = 24.68, P = 0.996). In addition, the MR-Egger regression test for the MR analysis of other outcomes revealed no horizontal pleiotropy and the Cochrane Q test revealed no heterogeneity (see Table 2). The leave-one-out analyses demonstrated the results’ consistency.

**Table 2:** Sensitivity analysis of the causal association between sleep traits and the risk of ESRD.

Exposure	Outcome	MR-IVW			MR-Egger			MR-Egger intercept			MR-PRESSO
		Q	Q_df	Q_pval	Q	Q_df	Q_pval	Intercept	SE	P val	Global test Pval
Sleeplessness/insomnia	ESRD	21.32	25	0.67	20.6	24	0.662	-0.06	0.07	0.4	0.782
Sleep duration		45.45	40	0.256	45.09	39	0.232	0.021	0.037	0.58	0.334
Morning chronotype		84.24	77	0.268	82.17	76	0.294	-0.024	0.017	0.17	0.201
Getting up in the morning		20.28	35	0.978	19.49	34	0.978	-0.025	0.028	0.38	0.976
Non-snoring		13.7	20	0.85	12.27	19	0.874	0.089	0.07	0.245	0.813
Daytime dozing		12.62	17	0.761	12.28	16	0.724	-0.053	0.09	0.567	0.783
Nap during the day		24.68	46	0.996	23.02	45	0.997	-0.046	0.036	0.204	0.997
ESRD	Sleeplessness/insomnia	18.72	19	0.475	18.56	18	0.419	-0.000575	0.00145	0.696	0.505
	Sleep duration	13.977	19	0.785	13.689	18	0.749	-0.0008	0.0015	0.598	0.805
	Morning chronotype	19.29	19	0.438	19.294	18	0.429	-0.0018	0.00198	0.367	0.378
	Getting up in the morning	12.44	19	0.866	10.28	18	0.922	0.0023	0.0015	0.159	0.834
	Non-snoring	32.58	19	0.027	29.83	18	0.039	0.0016	0.0012	0.214	0.616
	Daytime dozing	16.24	19	0.641	16.15	18	0.582	-0.00028	0.00098	0.777	0.624
	Nap during the day	24.69	19	0.17	18.97	18	0.335	-0.0026	0.0012	0.054	0.178
Sleeplessness/insomnia	Dialysis	36.41	26	0.084	28.78	25	0.273	-0.094	0.036	0.016	0.822
Sleep duration		37.87	40	0.567	36.44	39	0.587	0.047	0.039	0.239	0.604
Morning chronotype		97.76	79	0.075	97.26	78	0.069	0.013	0.019	0.528	0.094
Getting up in the morning		46.09	36	0.120	45.93	35	0.102	0.016	0.045	0.729	0.128
Non-snoring		37.39	21	0.015	36.27	20	0.014	-0.068	0.086	0.441	0.605
Daytime dozing		9.74	18	0.939	8.63	17	0.951	0.075	0.071	0.306	0.935
Nap during the day		56.12	47	0.169	56.08	46	0.147	-0.0075	0.038	0.846	0.148
Sleeplessness/insomnia	Glomerular filtration rate	15.37	10	0.119	15.12	9	0.088	0.0025	0.0065	0.706	0.103
Sleep duration		25.01	15	0.049	24.63	14	0.038	-0.0019	0.004	0.650	0.063
Morning chronotype		32.57	27	0.211	31.59	26	0.207	-0.0019	0.002	0.378	0.197
Getting up in the morning		30.22	15	0.011	30.169	14	0.007	-0.0006	0.0039	0.882	0.554
Non-snoring		18.68	10	0.044	17.67	9	0.039	0.0056	0.0079	0.492	0.054
Daytime dozing		19.15	9	0.023	18.94	8	0.015	0.0019	0.0063	0.775	0.317
Nap during the day		31.90	29	0.324	31.88	28	0.279	-0.00027	0.0019	0.888	0.368

ESRD, end-stage renal disease; OR, odds ratio; CI, confidence interval; IVW, inverse-variance weighted; FDR, P-value corrected for False Discovery Rate

### Reverse MR analysis

In assessing the influence of ESRD on the risk of sleep traits, the IVW technique revealed no evidence favouring a causal effect of ESRD on the risk of insomnia (OR = 0.997, 95%CI = 0.990–1.003, P = 0.324), getting up early (OR = 1.002, 95%CI 0.995–1.008, P = 0.616), non-snoring (OR = 1.002, 95%CI 0.995–1.010, P = 0.543), sleep duration (b = -0.001, 95%CI -0.008-0.006, P = 0.783), morning chronotype (OR = 0.994, 95%CI 0.986–1.003 P = 0.173), daytime napping (OR = 0.995, 95%CI 0.989–1.001 P = 0.088) or daytime dozing (OR = 1.001, 95%CI 0.996–1.006, P = 0.598). In the sensitivity analysis, the MR-Egger regression test revealed no horizontal pleiotropy and the Cochrane Q test revealed no heterogeneity (Table 2).

### Multivariable MR analysis

Genetic liability to diabetes and BMI was significantly associated with ESRD (See Supplementary Tables 10, Additional File 1). In the multivariable MR adjusting for diabetes, there was no evidence for a causal association of genetic liability to sleeplessness/ insomnia, getting up early in the morning and non-snoring with ESRD. Likewise, after adjustment for BMI, associations between genetic liability to sleeplessness/ insomnia, getting up early in the morning, and non-snoring and ESRD did not persist (See Supplementary Tables 11, Additional File1).

In the multivariable MR analysis adjusting for depression, getting up early in the morning consistently showed a causality with ESRD (OR = 0.246, 95%CI 0.093–0.647, P = 0.0045, FDR = 0.0114), and the association between non-snoring and ESRD remained significant (OR = 0.034, 95%CI 0.0028–0.407, P = 0.0076, FDR = 0.0114). However, the causal association between sleeplessness/ insomnia and ESRD disappeared. After adjusting for hypertension, most associations between sleep traits and ESRD did not persist, but only the association between the genetic reliability to non-snoring and ESRD remained significant (OR = 0.038, 95%CI 0.0029–0.512, P = 0.0135, FDR = 0.0405) (See Supplementary Tables 11, Additional File 1).

## Discussion

### Principal findings

In this bidirectional TSMR investigation, sleeplessness/insomnia, waking up early, and not snoring were suggestively associated with the risk of ESRD. Furthermore, However, our data did not show evidence providing a causal connection between genetic predisposition to ESRD and sleep disturbances. Some of the associations remained after adjustment for depression and hypertension. Diabetes and BMI might partly mediate the link between sleep traits and ESRD.

### Previous studies

Poor sleep quality and disruptions have been commonly observed in CKD and ESRD patients. A cross-sectional research indicated ESRD patients had higher sleep disturbances than CKD patients [29]. On the other hand, consistent with our findings, a recent observational research found that sleep disturbances were related to ESRD in patients with CKD [6] and that individuals with a healthy sleep pattern were associated with a substantial reduction in the risk of CKD [7].

In addition, we expanded these findings by demonstrating the effects of various sleep characteristics on ESRD. A Singapore Chinese Health Study found that both short and extended sleep durations were related to an increased risk of ESRD [30]. As for insomnia, a prospective cohort research revealed that nighttime insomnia is related to a modestly elevated risk of CKD [31], but a study reported that insomnia is not connected with the incidence of ESRD [32], which contradicts the present findings of MR analysis. There is a paucity of epidemiological research on healthy sleep habits, but a study indicated that a healthy lifestyle score system, which included a good sleep pattern, was connected with a reduced risk of CKD [33]. In addition, a study revealed that obstructive sleep apnea, the severity of which is related to snoring [34], contributed to the progression of CKD [35]. Although we identified a detrimental impact of insomnia on ESRD and that getting up in the morning and not snoring delayed disease development, there was inadequate previous evidence for causal associations between sleep traits and ESRD. Possible explanations include population differences, sample size, and inappropriate control of confounding variables.

According to research, sleep problems are linked to depression [36], which aligns with our discovery of a strong causal link between insomnia and depression. In addition, research suggested that depression may be a risk factor for the development of CKD [37]. This implied that sleep disturbance might affect ESRD through depression. The causal association between insomnia and ESRD attenuated after adjusting for depression, but the associations of non-snoring and getting up early in the morning with ESRD were even more robust in multivariable MR adjusting for depression, indicating that genetic correlations between these two sleep traits and depression are less likely to be a source of biassing these findings.

Additionally, consistent with our analysis of multivariable MR, sleep traits were shown to affect BMI [38], hypertension [39], and diabetes [40]. Several studies also linked BMI [41], hypertension [42], and type 2 diabetes [43] to ESRD. Specifically, our multivariable analysis linked sleep traits to obesity, hypertension, and an increased risk of diabetes. These factors may mediate the association between sleep traits and ESRD. After adjusting for these factors, most associations between sleep traits and ESRD did not persist, indicating that these factors might confound the observed associations between sleep traits and ESRD.

### Potential mechanisms

The precise pathophysiological mechanisms behind the link between sleep traits and ESRD remain poorly known. In this and previous research, sleep characteristics were connected to obesity, hypertension, and diabetes, and ESRD may be triggered by obesity [44], high blood pressure [42], and diabetes [43, 45].

Sleep disorders were associated with a condition of the hypothalamic-pituitary-adrenal axis [46]; snoring associated with OSA was linked to impaired autonomic nervous function [47]. The higher risk of ESRD in patients with insomnia may be partially explained by sleep-induced alterations in the autonomic nervous system and hypothalamic-pituitary axis [48, 49]. Additionally, inflammation might change due to insomnia, primary snoring, and obstructive sleep apnea [50, 51]. At the same time, systemic and local chronic inflammation (in the kidney) operate as risk factors for diabetic renal disease and its development into ESRD [52].

### Strengths and limitations

Our study has several strengths. This is the first MR analysis of sleep attributes with ESRD, probing evidence of the causal association between sleep-related characteristics and ESRD and studying the bidirectional causation relationship. Second, the MR design reduced the likelihood that confounding and other biases caused the observed bias. A large sample size and GWAS SNPs offered statistical validity for assessing causality. These steps improve conclusion validity.

Our study does, however, have certain shortcomings. First, participants in the ESRD where we obtained outcome data were Hispanic/Latino, while the exposure dataset we used was all European. A study showing genome-wide admixture mapping of CKD identified European ancestry-of-origin loci in Hispanic and Latino individuals, and the locus with European ancestry was associated with the CKD risk [53]. However, population stratification may contribute to confounders. The ancestry distribution, on the other hand, restricted the generalizability of our findings to other groups. Second, we could not determine if there were dose-response connections between sleep traits and ESRD. Still, uncertainty remains around the potential effects of public policy interventions on different sleep behaviors. Third, the findings of the Power analysis for non-snoring and getting up in the morning are minor, which might be due to the limited number of cases and sample size of ESRD. Finally, the results of the weighted median technique were not consistent with the suggestive associations provided by the IVW method in the primary MR analysis, indicating the presence of pleiotropy. Although we conducted sensitivity analyses that revealed no obvious pleiotropy, we find it difficult to verify the assumption that genetic instruments could only affect the outcome through the risk factor and not through pleiotropy.

## Conclusions

Overall, our study of MR analysis had no strong evidence to support a protective or deleterious effect of genetically predicted sleep traits on ESRD nor strong evidence to support an effect of ESRD on sleep disorders. However, this study provides suggestive associations between getting up early, insomnia, and non-snoring with ESRD, and the necessity of further research into the mechanism behind the link between sleep disorders and ESRD is highlighted by this study.

## Electronic supplementary material

Below is the link to the electronic supplementary material.

Supplementary Material 1

## Data Availability

GWAS summary statistics for sleep traits are publicly available through http://www.nealelab.is/blog/2017/7/19/rapid-gwas-of-thousands-of-phenotypes-for-337000-samples-in-the-uk-biobank. The summary statistics of GWAS for ESRD are derived from a GWAS conducted by Wojcik GL et al.(10.1038/s41586-019-1310-4). The data for dialysis is derived from https://www.finngen.fi/en. The summary data for the glomerular filtration rate is from a GWAS conducted by Pattaro C et al.( 10.1038/ncomms10023). In the assessment of risk factors, summary statistics for depression and hypertension are avalaible through https://www.finngen.fi/en. Summary statistics for body mass index (BMI) can be download from 10.7554/eLife.34408. The summary statistics of GWAS for diabetes are derived from a GWAS conducted by Xue A et al.( 10.1038/s41467-018-04951-w). All data can be downloaded from IEU OpenGWAS project (10.1101/2020.08.10.244293).

## Abbreviations

CKD: Chronic kidney disease
ESRD: End-stage renal disease
MR: Mendelian randomization
TSMR: Two-sample mendelian randomization
BMI: Body mass index
GWAS: Genome-wide association study
OR: Odds ratio
CI: Confidence interval
IVs: Instrumental variables
SNPs: Single nucleotide polymorphisms
MR-PRESSO: MR-pleiotropy residual sum and outlier
FDR: False discovery rate
PVE: phenotypic variation explained
