# Supplementary material for: Sleep traits and risk of end-stage renal disease: a mendelian randomization study
Source: BMC Med Genomics. 2023 Apr 7;16:76. doi: 10.1186/s12920-023-01497-9 (PMC10080763; doi:10.1186/s12920-023-01497-9)
Supplement: Supplementary file 1 — Supplementary Material 1 [file 12920_2023_1497_MOESM1_ESM.docx]

**Supplementary materials**

**The causal relationship between sleep traits and risk of end-stage kidney disease: a two-sample mendelian randomization study**

*Kaixin Li, Jiaxi Zhao, Wenjing Yang, Zhibin Ye*

**Supplementary Table 1.** Detailed information on included traits in the present study

| Phenotype | Year | Consortium | Sample size | Web link for data source |
| --- | --- | --- | --- | --- |
| Sleeplessness/insomnia | 2017 | Neale Lab | 336965 | http://www.nealelab.is/blog/2017/7/19/rapid-gwas-of-thousands-of-phenotypes-for-337000-samples-in-the-uk-biobank |
| Sleep duration | 2017 | Neale Lab | 335410 | http://www.nealelab.is/blog/2017/7/19/rapid-gwas-of-thousands-of-phenotypes-for-337000-samples-in-the-uk-biobank |
| Non-snoring | 2017 | Neale Lab | 314449 | http://www.nealelab.is/blog/2017/7/19/rapid-gwas-of-thousands-of-phenotypes-for-337000-samples-in-the-uk-biobank |
| Daytime dozing | 2017 | Neale Lab | 336082 | http://www.nealelab.is/blog/2017/7/19/rapid-gwas-of-thousands-of-phenotypes-for-337000-samples-in-the-uk-biobank |
| Nap during the day | 2017 | Neale Lab | 337074 | http://www.nealelab.is/blog/2017/7/19/rapid-gwas-of-thousands-of-phenotypes-for-337000-samples-in-the-uk-biobank |
| Getting up in the morning | 2017 | Neale Lab | 336501 | http://www.nealelab.is/blog/2017/7/19/rapid-gwas-of-thousands-of-phenotypes-for-337000-samples-in-the-uk-biobank |
| Morning' person (chronotype) | 2017 | Neale Lab | 301143 | http://www.nealelab.is/blog/2017/7/19/rapid-gwas-of-thousands-of-phenotypes-for-337000-samples-in-the-uk-biobank |
| End-stage renal disease | 2019 | NA | 33061 | https://www.ebi.ac.uk/gwas/downloads/summary-statistics |
| Depression | 2021 | FinnGen | 215644 | https://www.finngen.fi/en |
| Hypertension | 2021 | FinnGen | 218754 | https://www.finngen.fi/en |
| Body mass index | 2018 | GIANT | 681275 | https://gwas.mrcieu.ac.uk/datasets/ieu-b-40/ |
| Diabetes | 2018 | NA | 655666 | https://www.ebi.ac.uk/gwas/studies/GCST006867 |
| Dialysis | 2021 | FinnGen | 213489 | https://www.finngen.fi/en |
| Glumerular filtration rate | 2016 | NA | 32834 | https://www.ebi.ac.uk/gwas/studies/GCST003375 |

**Supplementary Table 2.** The summary information for instrumental variables of sleeplessness/ insomnia.

| Sort | Phenotype | SNP | effect_allele | other_allele | eaf | beta | se | pval |
| --- | --- | --- | --- | --- | --- | --- | --- | --- |
| 1 | Sleeplessness/insomnia | rs6690017 | G | T | 0.408 | -0.010 | 0.002 | 2.66E-08 |
| 2 | Sleeplessness/insomnia | rs11804386 | A | G | 0.333 | 0.010 | 0.002 | 3.59E-08 |
| 3 | Sleeplessness/insomnia | rs2644128 | G | C | 0.551 | 0.011 | 0.002 | 4.82E-10 |
| 4 | Sleeplessness/insomnia | rs7572387 | C | A | 0.429 | 0.011 | 0.002 | 6.21E-10 |
| 5 | Sleeplessness/insomnia | rs2863957 | A | C | 0.219 | -0.013 | 0.002 | 6.67E-10 |
| 6 | Sleeplessness/insomnia | rs113851554 | T | G | 0.057 | 0.048 | 0.004 | 1.16E-34 |
| 7 | Sleeplessness/insomnia | rs6744461 | C | A | 0.680 | 0.011 | 0.002 | 7.48E-09 |
| 8 | Sleeplessness/insomnia | rs4688760 | T | C | 0.692 | 0.012 | 0.002 | 1.06E-09 |
| 9 | Sleeplessness/insomnia | rs9878792 | T | G | 0.037 | -0.027 | 0.005 | 9.95E-09 |
| 10 | Sleeplessness/insomnia | rs9815484 | G | A | 0.818 | 0.013 | 0.002 | 5.64E-09 |
| 11 | Sleeplessness/insomnia | rs2132083 | C | T | 0.660 | -0.010 | 0.002 | 4.04E-08 |
| 12 | Sleeplessness/insomnia | rs1592757 | C | G | 0.356 | 0.010 | 0.002 | 1.24E-08 |
| 13 | Sleeplessness/insomnia | rs13186678 | T | C | 0.307 | 0.011 | 0.002 | 4.91E-09 |
| 14 | Sleeplessness/insomnia | rs3817576 | G | A | 0.525 | -0.010 | 0.002 | 2.81E-09 |
| 15 | Sleeplessness/insomnia | rs10280045 | G | C | 0.572 | 0.010 | 0.002 | 4.04E-08 |
| 16 | Sleeplessness/insomnia | rs10087341 | C | T | 0.159 | 0.015 | 0.002 | 7.74E-10 |
| 17 | Sleeplessness/insomnia | rs10156602 | G | A | 0.364 | -0.010 | 0.002 | 2.32E-08 |
| 18 | Sleeplessness/insomnia | rs224071 | A | G | 0.550 | 0.010 | 0.002 | 2.67E-08 |
| 19 | Sleeplessness/insomnia | rs3808937 | T | C | 0.208 | -0.014 | 0.002 | 2.59E-11 |
| 20 | Sleeplessness/insomnia | rs17879819 | T | C | 0.076 | -0.019 | 0.003 | 9.83E-09 |
| 21 | Sleeplessness/insomnia | rs2956278 | G | A | 0.214 | 0.012 | 0.002 | 2.03E-08 |
| 22 | Sleeplessness/insomnia | rs324017 | C | A | 0.705 | -0.012 | 0.002 | 8.83E-10 |
| 23 | Sleeplessness/insomnia | rs4886140 | G | A | 0.667 | 0.011 | 0.002 | 2.69E-09 |
| 24 | Sleeplessness/insomnia | rs4943439 | T | C | 0.387 | 0.010 | 0.002 | 4.75E-09 |
| 25 | Sleeplessness/insomnia | rs6561715 | A | T | 0.629 | -0.012 | 0.002 | 1.06E-11 |
| 26 | Sleeplessness/insomnia | rs1547630 | A | G | 0.652 | 0.010 | 0.002 | 1.23E-08 |
| 27 | Sleeplessness/insomnia | rs11635495 | C | T | 0.514 | 0.010 | 0.002 | 2.34E-08 |
| 28 | Sleeplessness/insomnia | rs1925501 | A | T | 0.220 | -0.012 | 0.002 | 1.54E-08 |
| 29 | Sleeplessness/insomnia | rs71373536 | A | G | 0.253 | 0.013 | 0.002 | 1.92E-11 |
| 30 | Sleeplessness/insomnia | rs11152363 | A | G | 0.185 | 0.014 | 0.002 | 2.54E-10 |

**Supplementary Table 3.** The summary information for instrumental variables of sleep duration.

| Sort | Phenotype | SNP | effect_allele | other_allele | eaf | beta | se | pval |
| --- | --- | --- | --- | --- | --- | --- | --- | --- |
| 1 | Sleep duration | rs4642942 | G | C | 0.586 | -0.012 | 0.002 | 2.67E-10 |
| 2 | Sleep duration | rs915416 | G | C | 0.710 | -0.012 | 0.002 | 2.53E-08 |
| 3 | Sleep duration | rs6681755 | A | G | 0.199 | 0.014 | 0.002 | 2.21E-09 |
| 4 | Sleep duration | rs12567114 | A | G | 0.275 | 0.013 | 0.002 | 2.71E-09 |
| 5 | Sleep duration | rs74889896 | G | A | 0.086 | 0.022 | 0.003 | 2.63E-11 |
| 6 | Sleep duration | rs4667876 | G | A | 0.448 | 0.011 | 0.002 | 2.89E-09 |
| 7 | Sleep duration | rs56337305 | C | T | 0.378 | 0.011 | 0.002 | 2.58E-08 |
| 8 | Sleep duration | rs374153 | T | C | 0.841 | -0.015 | 0.003 | 9.35E-09 |
| 9 | Sleep duration | rs10496079 | C | G | 0.629 | 0.015 | 0.002 | 1.44E-14 |
| 10 | Sleep duration | rs80071665 | T | G | 0.120 | -0.016 | 0.003 | 1.75E-08 |
| 11 | Sleep duration | rs62158206 | C | T | 0.219 | 0.030 | 0.002 | 1.33E-39 |
| 12 | Sleep duration | rs113021516 | C | G | 0.336 | 0.011 | 0.002 | 2.79E-08 |
| 13 | Sleep duration | rs9810474 | T | C | 0.233 | -0.013 | 0.002 | 8.74E-09 |
| 14 | Sleep duration | rs12501164 | C | T | 0.497 | 0.011 | 0.002 | 2.05E-08 |
| 15 | Sleep duration | rs13107325 | T | C | 0.075 | -0.022 | 0.004 | 9.34E-10 |
| 16 | Sleep duration | rs180769 | C | T | 0.576 | -0.011 | 0.002 | 7.49E-09 |
| 17 | Sleep duration | rs6889592 | A | G | 0.335 | 0.011 | 0.002 | 3.23E-08 |
| 18 | Sleep duration | rs37021 | G | A | 0.443 | -0.011 | 0.002 | 2.86E-08 |
| 19 | Sleep duration | rs7764984 | G | A | 0.327 | -0.013 | 0.002 | 8.84E-11 |
| 20 | Sleep duration | rs4897409 | A | G | 0.305 | -0.011 | 0.002 | 4.50E-08 |
| 21 | Sleep duration | rs62444917 | C | A | 0.220 | 0.013 | 0.002 | 1.78E-08 |
| 22 | Sleep duration | rs4730640 | C | T | 0.616 | -0.013 | 0.002 | 1.69E-11 |
| 23 | Sleep duration | rs11982852 | T | C | 0.245 | -0.013 | 0.002 | 1.43E-09 |
| 24 | Sleep duration | rs448231 | A | T | 0.444 | 0.011 | 0.002 | 1.62E-09 |
| 25 | Sleep duration | rs7016314 | C | T | 0.657 | 0.011 | 0.002 | 1.22E-08 |
| 26 | Sleep duration | rs28375265 | T | A | 0.348 | -0.011 | 0.002 | 4.15E-08 |
| 27 | Sleep duration | rs4588900 | A | G | 0.517 | -0.010 | 0.002 | 2.84E-08 |
| 28 | Sleep duration | rs10973207 | T | G | 0.159 | 0.014 | 0.003 | 2.87E-08 |
| 29 | Sleep duration | rs1570203 | A | G | 0.525 | -0.012 | 0.002 | 1.90E-10 |
| 30 | Sleep duration | rs10510128 | A | G | 0.206 | 0.014 | 0.002 | 9.17E-10 |
| 31 | Sleep duration | rs925872 | G | C | 0.455 | -0.011 | 0.002 | 1.46E-09 |
| 32 | Sleep duration | rs61916239 | G | T | 0.350 | 0.011 | 0.002 | 4.26E-08 |
| 33 | Sleep duration | rs35186585 | C | A | 0.229 | -0.013 | 0.002 | 1.55E-08 |
| 34 | Sleep duration | rs7115462 | A | G | 0.073 | 0.021 | 0.004 | 4.85E-09 |
| 35 | Sleep duration | rs4767550 | G | A | 0.415 | 0.011 | 0.002 | 3.72E-08 |
| 36 | Sleep duration | rs11621908 | T | C | 0.082 | -0.019 | 0.003 | 3.23E-08 |
| 37 | Sleep duration | rs8029928 | T | C | 0.239 | -0.014 | 0.002 | 2.58E-10 |
| 38 | Sleep duration | rs3751813 | T | G | 0.542 | -0.012 | 0.002 | 9.04E-10 |
| 39 | Sleep duration | rs11643715 | G | C | 0.290 | 0.011 | 0.002 | 3.01E-08 |
| 40 | Sleep duration | rs802310 | G | A | 0.212 | 0.015 | 0.002 | 1.18E-10 |
| 41 | Sleep duration | rs17822558 | A | G | 0.323 | -0.011 | 0.002 | 2.51E-08 |
| 42 | Sleep duration | rs9895274 | T | C | 0.488 | -0.010 | 0.002 | 3.87E-08 |
| 43 | Sleep duration | rs11650677 | A | G | 0.338 | 0.012 | 0.002 | 5.85E-09 |
| 44 | Sleep duration | rs34786000 | T | G | 0.556 | 0.012 | 0.002 | 3.90E-10 |

**Supplementary Table 4.** The summary information for instrumental variables of getting up in the morning.

| Sort | Phenotype | SNP | effect_allele | other_allele | eaf | beta | se | pval |
| --- | --- | --- | --- | --- | --- | --- | --- | --- |
| 1 | getting up in the morning | rs12752290 | C | T | 0.442 | 0.011 | 0.002 | 3.19E-09 |
| 2 | getting up in the morning | rs12736689 | C | T | 0.030 | 0.052 | 0.005 | 6.62E-22 |
| 3 | getting up in the morning | rs10518446 | C | G | 0.164 | 0.021 | 0.003 | 6.84E-17 |
| 4 | getting up in the morning | rs6666373 | T | A | 0.272 | 0.011 | 0.002 | 4.27E-08 |
| 5 | getting up in the morning | rs10462020 | G | T | 0.197 | 0.015 | 0.002 | 3.70E-10 |
| 6 | getting up in the morning | rs78555397 | T | C | 0.030 | 0.038 | 0.005 | 3.04E-12 |
| 7 | getting up in the morning | rs76048411 | C | T | 0.518 | -0.011 | 0.002 | 1.46E-09 |
| 8 | getting up in the morning | rs1542344 | G | A | 0.666 | 0.012 | 0.002 | 5.32E-10 |
| 9 | getting up in the morning | rs10175975 | T | C | 0.180 | 0.013 | 0.002 | 5.00E-08 |
| 10 | getting up in the morning | rs9309511 | G | A | 0.427 | -0.016 | 0.002 | 6.28E-17 |
| 11 | getting up in the morning | rs1039887 | G | A | 0.151 | -0.020 | 0.003 | 4.16E-14 |
| 12 | getting up in the morning | rs1402121 | C | T | 0.200 | 0.014 | 0.002 | 3.44E-09 |
| 13 | getting up in the morning | rs2859580 | A | G | 0.886 | 0.017 | 0.003 | 5.72E-09 |
| 14 | getting up in the morning | rs4958318 | T | C | 0.289 | 0.014 | 0.002 | 2.16E-12 |
| 15 | getting up in the morning | rs4716098 | G | A | 0.075 | -0.019 | 0.004 | 4.12E-08 |
| 16 | getting up in the morning | rs2653356 | G | A | 0.820 | -0.024 | 0.002 | 9.95E-24 |
| 17 | getting up in the morning | rs72895663 | G | A | 0.233 | 0.012 | 0.002 | 2.62E-08 |
| 18 | getting up in the morning | rs620598 | G | A | 0.225 | 0.013 | 0.002 | 6.66E-09 |
| 19 | getting up in the morning | rs1914397 | A | T | 0.449 | 0.011 | 0.002 | 1.81E-08 |
| 20 | getting up in the morning | rs1564470 | A | C | 0.332 | 0.011 | 0.002 | 2.83E-08 |
| 21 | getting up in the morning | rs2360802 | T | A | 0.226 | 0.013 | 0.002 | 1.21E-08 |
| 22 | getting up in the morning | rs117965994 | T | C | 0.043 | -0.025 | 0.005 | 3.34E-08 |
| 23 | getting up in the morning | rs77641763 | T | C | 0.125 | -0.019 | 0.003 | 8.04E-12 |
| 24 | getting up in the morning | rs10760663 | A | G | 0.551 | 0.011 | 0.002 | 5.44E-09 |
| 25 | getting up in the morning | rs4962716 | C | T | 0.872 | 0.016 | 0.003 | 3.07E-08 |
| 26 | getting up in the morning | rs11181153 | T | C | 0.586 | -0.010 | 0.002 | 3.32E-08 |
| 27 | getting up in the morning | rs17464772 | A | G | 0.355 | 0.014 | 0.002 | 4.89E-12 |
| 28 | getting up in the morning | rs4964519 | G | C | 0.626 | 0.011 | 0.002 | 1.79E-08 |
| 29 | getting up in the morning | rs12227309 | T | C | 0.246 | 0.013 | 0.002 | 3.34E-09 |
| 30 | getting up in the morning | rs57140837 | C | T | 0.303 | 0.012 | 0.002 | 9.67E-09 |
| 31 | getting up in the morning | rs1014895 | T | G | 0.386 | 0.011 | 0.002 | 1.06E-08 |
| 32 | getting up in the morning | rs6495249 | T | C | 0.381 | 0.012 | 0.002 | 1.55E-09 |
| 33 | getting up in the morning | rs2908897 | T | G | 0.702 | -0.013 | 0.002 | 8.03E-10 |
| 34 | getting up in the morning | rs1421085 | C | T | 0.402 | 0.011 | 0.002 | 2.92E-08 |
| 35 | getting up in the morning | rs2532317 | C | T | 0.204 | 0.015 | 0.003 | 1.88E-09 |
| 36 | getting up in the morning | rs77556405 | A | G | 0.174 | 0.016 | 0.002 | 1.07E-10 |
| 37 | getting up in the morning | rs34626694 | T | C | 0.331 | -0.011 | 0.002 | 1.29E-08 |
| 38 | getting up in the morning | rs4790352 | A | G | 0.918 | 0.020 | 0.003 | 5.34E-09 |
| 39 | getting up in the morning | rs12969848 | T | C | 0.530 | 0.011 | 0.002 | 1.76E-08 |
| 40 | getting up in the morning | rs13058062 | C | T | 0.713 | -0.011 | 0.002 | 3.86E-08 |

**Supplementary Table 5.** The summary information for instrumental variables of chronotype.

| Sort | Phenotype | SNP | effect_allele | other_allele | eaf | se | beta | pval |
| --- | --- | --- | --- | --- | --- | --- | --- | --- |
| 1 | Chronotype | rs139315125 | G | A | 0.005 | 0.017 | -0.153 | 1.00E-18 |
| 2 | Chronotype | rs11587758 | A | G | 0.394 | 0.002 | -0.018 | 4.06E-14 |
| 3 | Chronotype | rs12040629 | A | G | 0.161 | 0.003 | -0.030 | 1.06E-20 |
| 4 | Chronotype | rs228674 | T | C | 0.097 | 0.004 | 0.026 | 1.99E-10 |
| 5 | Chronotype | rs17416732 | C | T | 0.374 | 0.002 | -0.015 | 1.05E-09 |
| 6 | Chronotype | rs12140153 | T | G | 0.097 | 0.004 | 0.028 | 7.67E-12 |
| 7 | Chronotype | rs975025 | T | C | 0.077 | 0.004 | 0.025 | 2.69E-08 |
| 8 | Chronotype | rs61770703 | C | T | 0.520 | 0.002 | -0.014 | 6.93E-09 |
| 9 | Chronotype | rs72720396 | G | A | 0.231 | 0.003 | -0.021 | 5.66E-14 |
| 10 | Chronotype | rs509476 | C | T | 0.970 | 0.007 | 0.082 | 4.46E-32 |
| 11 | Chronotype | rs778147 | A | C | 0.634 | 0.002 | 0.015 | 7.50E-10 |
| 12 | Chronotype | rs80271258 | T | C | 0.088 | 0.004 | 0.042 | 6.43E-23 |
| 13 | Chronotype | rs113851554 | T | G | 0.057 | 0.005 | 0.030 | 2.17E-08 |
| 14 | Chronotype | rs10520176 | C | T | 0.498 | 0.002 | 0.021 | 6.10E-18 |
| 15 | Chronotype | rs10189912 | G | A | 0.370 | 0.002 | 0.018 | 1.33E-13 |
| 16 | Chronotype | rs1947198 | T | C | 0.122 | 0.004 | -0.020 | 2.10E-08 |
| 17 | Chronotype | rs4417706 | T | A | 0.483 | 0.002 | -0.015 | 1.53E-10 |
| 18 | Chronotype | rs13011556 | G | C | 0.239 | 0.003 | -0.017 | 9.71E-10 |
| 19 | Chronotype | rs1067404 | G | T | 0.758 | 0.003 | -0.020 | 1.08E-12 |
| 20 | Chronotype | rs10495976 | T | A | 0.392 | 0.002 | -0.014 | 8.24E-09 |
| 21 | Chronotype | rs850894 | T | A | 0.707 | 0.003 | 0.015 | 3.28E-08 |
| 22 | Chronotype | rs13316611 | T | G | 0.256 | 0.003 | -0.016 | 4.13E-09 |
| 23 | Chronotype | rs75815271 | C | T | 0.074 | 0.005 | -0.026 | 1.86E-08 |
| 24 | Chronotype | rs9831488 | G | A | 0.349 | 0.003 | -0.017 | 1.87E-11 |
| 25 | Chronotype | rs4685141 | T | C | 0.130 | 0.004 | 0.020 | 2.80E-08 |
| 26 | Chronotype | rs2125109 | T | C | 0.307 | 0.003 | 0.016 | 1.96E-09 |
| 27 | Chronotype | rs66710942 | T | C | 0.593 | 0.002 | -0.015 | 2.80E-09 |
| 28 | Chronotype | rs2239626 | C | T | 0.303 | 0.003 | -0.016 | 4.49E-10 |
| 29 | Chronotype | rs1107268 | G | A | 0.404 | 0.002 | 0.013 | 3.62E-08 |
| 30 | Chronotype | rs6846730 | T | C | 0.231 | 0.003 | 0.016 | 4.34E-08 |
| 31 | Chronotype | rs1296328 | C | A | 0.560 | 0.002 | 0.016 | 6.48E-11 |
| 32 | Chronotype | rs304136 | C | T | 0.477 | 0.002 | 0.015 | 4.57E-10 |
| 33 | Chronotype | rs67988891 | G | C | 0.319 | 0.003 | -0.018 | 2.69E-12 |
| 34 | Chronotype | rs9348050 | C | T | 0.511 | 0.002 | 0.014 | 6.39E-09 |
| 35 | Chronotype | rs9381812 | G | A | 0.297 | 0.003 | -0.017 | 2.91E-11 |
| 36 | Chronotype | rs2653349 | G | A | 0.784 | 0.003 | 0.027 | 2.82E-20 |
| 37 | Chronotype | rs75284686 | T | C | 0.010 | 0.012 | -0.086 | 5.45E-13 |
| 38 | Chronotype | rs9278039 | C | T | 0.143 | 0.003 | 0.019 | 2.84E-08 |
| 39 | Chronotype | rs60616179 | G | A | 0.055 | 0.005 | 0.033 | 3.80E-10 |
| 40 | Chronotype | rs10280205 | C | T | 0.307 | 0.003 | 0.015 | 7.59E-09 |
| 41 | Chronotype | rs4729303 | T | C | 0.814 | 0.003 | -0.021 | 3.11E-12 |
| 42 | Chronotype | rs4729854 | A | T | 0.487 | 0.002 | 0.022 | 5.13E-19 |
| 43 | Chronotype | rs7783012 | A | G | 0.592 | 0.002 | -0.014 | 9.67E-09 |
| 44 | Chronotype | rs2971970 | G | T | 0.782 | 0.003 | 0.017 | 6.09E-09 |
| 45 | Chronotype | rs6967481 | T | C | 0.497 | 0.002 | -0.017 | 5.28E-13 |
| 46 | Chronotype | rs56265640 | A | G | 0.233 | 0.003 | -0.017 | 2.96E-09 |
| 47 | Chronotype | rs9987253 | A | G | 0.221 | 0.003 | 0.018 | 1.90E-10 |
| 48 | Chronotype | rs16881183 | T | C | 0.377 | 0.002 | -0.014 | 1.07E-08 |
| 49 | Chronotype | rs17716502 | T | C | 0.207 | 0.003 | -0.019 | 3.81E-10 |
| 50 | Chronotype | rs117823615 | G | C | 0.085 | 0.004 | 0.026 | 9.49E-10 |
| 51 | Chronotype | rs28458909 | T | C | 0.125 | 0.004 | 0.030 | 1.53E-16 |
| 52 | Chronotype | rs6477309 | T | C | 0.666 | 0.003 | -0.014 | 2.65E-08 |
| 53 | Chronotype | rs77598468 | A | C | 0.035 | 0.006 | 0.039 | 2.69E-09 |
| 54 | Chronotype | rs10825272 | A | G | 0.145 | 0.003 | 0.020 | 4.27E-09 |
| 55 | Chronotype | rs11032362 | A | G | 0.090 | 0.004 | -0.024 | 1.30E-08 |
| 56 | Chronotype | rs11218901 | T | C | 0.136 | 0.003 | 0.019 | 4.69E-08 |
| 57 | Chronotype | rs7111355 | A | G | 0.837 | 0.003 | 0.018 | 3.83E-08 |
| 58 | Chronotype | rs11605297 | A | G | 0.233 | 0.003 | 0.017 | 2.13E-09 |
| 59 | Chronotype | rs73325957 | C | T | 0.140 | 0.003 | 0.019 | 3.50E-08 |
| 60 | Chronotype | rs11181153 | T | C | 0.586 | 0.002 | 0.017 | 7.34E-13 |
| 61 | Chronotype | rs7313852 | A | G | 0.435 | 0.002 | 0.022 | 1.06E-19 |
| 62 | Chronotype | rs73208536 | G | A | 0.395 | 0.002 | -0.015 | 1.62E-09 |
| 63 | Chronotype | rs80097534 | T | G | 0.099 | 0.004 | 0.023 | 7.21E-09 |
| 64 | Chronotype | rs7302062 | C | T | 0.448 | 0.002 | 0.013 | 1.95E-08 |
| 65 | Chronotype | rs7308565 | T | C | 0.401 | 0.002 | -0.015 | 9.05E-10 |
| 66 | Chronotype | rs7316768 | T | G | 0.403 | 0.002 | 0.015 | 1.80E-09 |
| 67 | Chronotype | rs2762088 | G | T | 0.760 | 0.003 | -0.015 | 4.60E-08 |
| 68 | Chronotype | rs2322000 | T | A | 0.321 | 0.003 | -0.015 | 2.40E-09 |
| 69 | Chronotype | rs7317373 | C | T | 0.034 | 0.007 | 0.055 | 4.89E-17 |
| 70 | Chronotype | rs1958885 | G | T | 0.729 | 0.003 | 0.015 | 3.68E-08 |
| 71 | Chronotype | rs4899502 | A | G | 0.689 | 0.003 | 0.014 | 4.58E-08 |
| 72 | Chronotype | rs12432176 | A | C | 0.380 | 0.002 | -0.015 | 1.15E-09 |
| 73 | Chronotype | rs1421085 | C | T | 0.402 | 0.002 | -0.022 | 6.32E-20 |
| 74 | Chronotype | rs1190652 | G | T | 0.920 | 0.004 | -0.024 | 4.32E-08 |
| 75 | Chronotype | rs17604349 | A | G | 0.177 | 0.003 | 0.019 | 6.52E-10 |
| 76 | Chronotype | rs12927162 | G | A | 0.277 | 0.003 | 0.021 | 1.60E-15 |
| 77 | Chronotype | rs4784655 | C | G | 0.321 | 0.003 | 0.015 | 9.90E-09 |
| 78 | Chronotype | rs2364972 | G | A | 0.465 | 0.002 | -0.013 | 4.44E-08 |
| 79 | Chronotype | rs4792268 | A | G | 0.920 | 0.004 | 0.034 | 1.51E-14 |
| 80 | Chronotype | rs2232839 | T | C | 0.218 | 0.003 | 0.019 | 3.54E-11 |
| 81 | Chronotype | rs12949341 | G | A | 0.331 | 0.003 | 0.014 | 2.26E-08 |
| 82 | Chronotype | rs62082401 | G | C | 0.190 | 0.003 | -0.022 | 7.42E-13 |
| 83 | Chronotype | rs4527088 | G | A | 0.411 | 0.002 | 0.017 | 2.04E-12 |
| 84 | Chronotype | rs9964420 | A | C | 0.305 | 0.003 | 0.021 | 1.00E-15 |
| 85 | Chronotype | rs6131942 | G | A | 0.579 | 0.002 | -0.013 | 2.88E-08 |
| 86 | Chronotype | rs117143374 | C | T | 0.142 | 0.003 | 0.020 | 1.09E-08 |
| 87 | Chronotype | rs139911 | T | C | 0.576 | 0.002 | 0.019 | 1.57E-14 |

**Supplementary Table 6.** The summary information for instrumental variables of napping during the day.

| Sort | Phenotype | SNP | effect_allele | other_allele | se | eaf | beta | pval |
| --- | --- | --- | --- | --- | --- | --- | --- | --- |
| 1 | Nap during the day | rs60579048 | G | T | 0.002 | 0.168 | -0.012 | 3.39E-10 |
| 2 | Nap during the day | rs12140153 | T | G | 0.002 | 0.097 | -0.027 | 7.87E-27 |
| 3 | Nap during the day | rs1843815 | T | A | 0.001 | 0.546 | 0.008 | 2.23E-08 |
| 4 | Nap during the day | rs35039375 | G | A | 0.003 | 0.092 | 0.014 | 8.18E-09 |
| 5 | Nap during the day | rs2250377 | G | A | 0.002 | 0.662 | -0.015 | 6.82E-24 |
| 6 | Nap during the day | rs17369061 | C | T | 0.002 | 0.128 | -0.012 | 4.77E-08 |
| 7 | Nap during the day | rs4971718 | T | C | 0.001 | 0.502 | 0.009 | 3.85E-10 |
| 8 | Nap during the day | rs2390669 | C | A | 0.002 | 0.129 | -0.013 | 6.91E-09 |
| 9 | Nap during the day | rs11125776 | G | T | 0.002 | 0.146 | -0.015 | 8.96E-13 |
| 10 | Nap during the day | rs12615434 | T | C | 0.002 | 0.113 | 0.013 | 2.16E-08 |
| 11 | Nap during the day | rs908442 | T | A | 0.001 | 0.408 | -0.011 | 5.31E-14 |
| 12 | Nap during the day | rs13033444 | G | A | 0.002 | 0.283 | 0.011 | 1.47E-11 |
| 13 | Nap during the day | rs253662 | C | T | 0.002 | 0.810 | 0.010 | 1.47E-08 |
| 14 | Nap during the day | rs12506659 | T | C | 0.001 | 0.486 | -0.009 | 7.01E-10 |
| 15 | Nap during the day | rs6854055 | T | G | 0.002 | 0.747 | 0.012 | 1.71E-13 |
| 16 | Nap during the day | rs1968557 | T | C | 0.001 | 0.495 | -0.008 | 2.82E-08 |
| 17 | Nap during the day | rs2431108 | C | T | 0.002 | 0.329 | 0.013 | 8.10E-18 |
| 18 | Nap during the day | rs10875622 | A | G | 0.001 | 0.576 | 0.010 | 1.89E-11 |
| 19 | Nap during the day | rs742787 | C | T | 0.001 | 0.370 | -0.010 | 6.65E-12 |
| 20 | Nap during the day | rs12193281 | C | T | 0.002 | 0.331 | -0.010 | 1.58E-10 |
| 21 | Nap during the day | rs2653349 | G | A | 0.002 | 0.784 | -0.017 | 1.36E-21 |
| 22 | Nap during the day | rs614987 | C | A | 0.001 | 0.615 | 0.012 | 3.82E-16 |
| 23 | Nap during the day | rs11761181 | C | T | 0.002 | 0.229 | 0.011 | 2.92E-10 |
| 24 | Nap during the day | rs12535424 | A | G | 0.003 | 0.087 | -0.015 | 1.28E-08 |
| 25 | Nap during the day | rs285793 | A | G | 0.001 | 0.538 | -0.009 | 2.08E-09 |
| 26 | Nap during the day | rs2555571 | G | T | 0.001 | 0.520 | -0.008 | 4.43E-08 |
| 27 | Nap during the day | rs11138082 | C | T | 0.002 | 0.205 | 0.014 | 2.19E-15 |
| 28 | Nap during the day | rs12682981 | G | A | 0.002 | 0.254 | -0.010 | 9.91E-10 |
| 29 | Nap during the day | rs17502738 | C | T | 0.002 | 0.196 | -0.010 | 1.58E-08 |
| 30 | Nap during the day | rs11258652 | A | C | 0.002 | 0.238 | -0.010 | 3.01E-09 |
| 31 | Nap during the day | rs10840017 | G | A | 0.002 | 0.230 | -0.010 | 1.14E-08 |
| 32 | Nap during the day | rs1836124 | T | C | 0.002 | 0.299 | -0.009 | 3.96E-08 |
| 33 | Nap during the day | rs174541 | C | T | 0.001 | 0.361 | 0.010 | 1.28E-11 |
| 34 | Nap during the day | rs35011311 | T | G | 0.002 | 0.267 | -0.009 | 4.69E-08 |
| 35 | Nap during the day | rs1983336 | A | G | 0.001 | 0.405 | 0.018 | 5.08E-34 |
| 36 | Nap during the day | rs2769916 | A | G | 0.002 | 0.689 | 0.009 | 4.45E-08 |
| 37 | Nap during the day | rs12147887 | T | C | 0.002 | 0.185 | 0.011 | 5.65E-09 |
| 38 | Nap during the day | rs2370926 | C | T | 0.001 | 0.367 | -0.008 | 2.45E-08 |
| 39 | Nap during the day | rs17158413 | A | G | 0.002 | 0.239 | 0.010 | 7.71E-10 |
| 40 | Nap during the day | rs114488427 | T | C | 0.002 | 0.120 | 0.012 | 2.92E-08 |
| 41 | Nap during the day | rs8050478 | A | G | 0.001 | 0.500 | -0.008 | 3.55E-08 |
| 42 | Nap during the day | rs2668643 | A | C | 0.002 | 0.226 | -0.021 | 8.52E-35 |
| 43 | Nap during the day | rs112520848 | C | G | 0.001 | 0.386 | 0.008 | 4.77E-08 |
| 44 | Nap during the day | rs3935190 | A | G | 0.001 | 0.534 | 0.009 | 1.72E-09 |
| 45 | Nap during the day | rs12451365 | C | T | 0.002 | 0.205 | 0.011 | 5.60E-10 |
| 46 | Nap during the day | rs2033103 | T | C | 0.001 | 0.452 | 0.009 | 2.01E-09 |
| 47 | Nap during the day | rs962247 | A | G | 0.001 | 0.477 | -0.009 | 3.55E-10 |
| 48 | Nap during the day | rs17723754 | T | C | 0.001 | 0.427 | -0.014 | 4.75E-21 |
| 49 | Nap during the day | rs3810484 | G | A | 0.001 | 0.444 | -0.008 | 2.68E-08 |
| 50 | Nap during the day | rs2836918 | C | T | 0.002 | 0.285 | 0.009 | 1.37E-08 |

**Supplementary Table 7.** The summary information for instrumental variables of non-snoring.

| Sort | Phenotype | SNP | effect_allele | other_allele | eaf | se | beta | pval |
| --- | --- | --- | --- | --- | --- | --- | --- | --- |
| 1 | Non-snoring | rs12128472 | G | C | 0.078 | 0.002 | -0.013 | 2.53E-09 |
| 2 | Non-snoring | rs61597598 | A | G | 0.136 | 0.002 | -0.012 | 4.63E-12 |
| 3 | Non-snoring | rs9309771 | G | A | 0.547 | 0.001 | 0.008 | 3.22E-10 |
| 4 | Non-snoring | rs34811474 | A | G | 0.232 | 0.001 | 0.009 | 1.81E-09 |
| 5 | Non-snoring | rs10062026 | A | G | 0.362 | 0.001 | 0.007 | 2.14E-08 |
| 6 | Non-snoring | rs2307111 | C | T | 0.393 | 0.001 | 0.007 | 9.52E-09 |
| 7 | Non-snoring | rs4523230 | T | A | 0.715 | 0.001 | -0.007 | 1.92E-08 |
| 8 | Non-snoring | rs13251292 | G | A | 0.412 | 0.001 | -0.008 | 4.97E-11 |
| 9 | Non-snoring | rs1775550 | A | G | 0.812 | 0.002 | 0.009 | 2.21E-09 |
| 10 | Non-snoring | rs7930256 | C | T | 0.356 | 0.001 | -0.007 | 1.70E-08 |
| 11 | Non-snoring | rs11041980 | A | T | 0.447 | 0.001 | 0.008 | 5.53E-11 |
| 12 | Non-snoring | rs10878271 | C | T | 0.635 | 0.001 | 0.008 | 1.28E-09 |
| 13 | Non-snoring | rs10505911 | A | C | 0.219 | 0.001 | -0.008 | 3.72E-08 |
| 14 | Non-snoring | rs592333 | G | A | 0.443 | 0.001 | -0.009 | 1.01E-13 |
| 15 | Non-snoring | rs9515311 | T | C | 0.368 | 0.001 | 0.007 | 1.63E-08 |
| 16 | Non-snoring | rs2614464 | A | G | 0.423 | 0.001 | 0.008 | 1.10E-10 |
| 17 | Non-snoring | rs12925525 | T | G | 0.068 | 0.002 | -0.013 | 2.07E-08 |
| 18 | Non-snoring | rs11075985 | A | C | 0.423 | 0.001 | -0.007 | 1.11E-09 |
| 19 | Non-snoring | rs1108431 | T | C | 0.373 | 0.001 | -0.008 | 7.61E-11 |
| 20 | Non-snoring | rs62066451 | G | A | 0.051 | 0.003 | 0.017 | 2.17E-09 |
| 21 | Non-snoring | rs199497 | C | T | 0.175 | 0.002 | -0.010 | 2.48E-10 |
| 22 | Non-snoring | rs12449873 | C | G | 0.376 | 0.001 | 0.007 | 1.74E-09 |
| 23 | Non-snoring | rs1641511 | A | G | 0.760 | 0.001 | 0.008 | 1.62E-08 |

**Supplementary Table 8.** The summary information for instrumental variables of daytime dozing.

| Sort | Phenotype | SNP | effect_allele | other_allele | eaf | se | beta | pval |
| --- | --- | --- | --- | --- | --- | --- | --- | --- |
| 1 | Daytime dozing | rs811483 | T | C | 0.168 | 0.002 | -0.009 | 7.52E-09 |
| 2 | Daytime dozing | rs12140153 | T | G | 0.097 | 0.002 | -0.017 | 9.87E-17 |
| 3 | Daytime dozing | rs553314 | C | T | 0.634 | 0.001 | -0.007 | 3.70E-09 |
| 4 | Daytime dozing | rs780093 | C | T | 0.619 | 0.001 | -0.008 | 8.62E-11 |
| 5 | Daytime dozing | rs13023284 | C | T | 0.604 | 0.001 | -0.008 | 4.15E-11 |
| 6 | Daytime dozing | rs13010456 | G | A | 0.406 | 0.001 | -0.009 | 2.21E-13 |
| 7 | Daytime dozing | rs843372 | T | C | 0.770 | 0.001 | -0.008 | 1.37E-08 |
| 8 | Daytime dozing | rs4242242 | A | G | 0.419 | 0.001 | -0.007 | 2.01E-09 |
| 9 | Daytime dozing | rs10900858 | G | A | 0.527 | 0.001 | -0.007 | 3.18E-08 |
| 10 | Daytime dozing | rs614987 | C | A | 0.615 | 0.001 | 0.007 | 2.71E-08 |
| 11 | Daytime dozing | rs3122170 | A | C | 0.768 | 0.001 | -0.009 | 2.24E-10 |
| 12 | Daytime dozing | rs6923811 | C | T | 0.321 | 0.001 | -0.007 | 2.39E-08 |
| 13 | Daytime dozing | rs35284403 | C | T | 0.350 | 0.001 | 0.007 | 8.83E-09 |
| 14 | Daytime dozing | rs285793 | A | G | 0.538 | 0.001 | -0.007 | 6.33E-09 |
| 15 | Daytime dozing | rs13284688 | C | T | 0.206 | 0.001 | 0.010 | 4.75E-12 |
| 16 | Daytime dozing | rs7476897 | A | G | 0.321 | 0.001 | -0.008 | 4.87E-09 |
| 17 | Daytime dozing | rs1846644 | C | T | 0.411 | 0.001 | 0.011 | 1.96E-18 |
| 18 | Daytime dozing | rs17356118 | G | A | 0.232 | 0.001 | 0.008 | 2.69E-08 |
| 19 | Daytime dozing | rs2696532 | G | A | 0.223 | 0.001 | -0.009 | 2.61E-10 |

**Supplementary Table 9.** The summary information for instrumental variables of ESRD

| Sort | Phenotype | SNP | effect_allele | other_allele | eaf | beta | se | pval |
| --- | --- | --- | --- | --- | --- | --- | --- | --- |
| 1 | End-stage renal disease | rs73044536 | G | C | 0.315096 | 0.220557 | 0.04751 | 3.45E-06 |
| 2 | End-stage renal disease | rs9847000 | G | A | 0.278169 | -0.22656 | 0.048911 | 3.62E-06 |
| 3 | End-stage renal disease | rs9469220 | A | G | 0.580181 | -0.21229 | 0.044968 | 2.35E-06 |
| 4 | End-stage renal disease | rs6906363 | C | T | 0.727665 | -0.23543 | 0.049317 | 1.81E-06 |
| 5 | End-stage renal disease | rs8050506 | A | G | 0.210795 | 0.249872 | 0.051069 | 9.94E-07 |
| 6 | End-stage renal disease | rs738329 | C | T | 0.707155 | 0.294403 | 0.060622 | 1.20E-06 |
| 7 | End-stage renal disease | rs136160 | G | C | 0.684003 | -0.2319 | 0.048857 | 2.07E-06 |

**Supplementary Table 10.** Associations of genetically predicted liability to sleep traits and ESRD with risk factors.

| **Exposure** | **Outcome** | **IVW** | | |
| --- | --- | --- | --- | --- |
|  |  | OR | 95%CI | P |
| **Insomnia** | Body mass index | 1.243 | 1.008-1.532 | 0.042* |
|  | Depression | 1.91 | 1.25-2.92 | 0.0029* |
|  | Hypertension | 1.438 | 0.817-2.532 | 0.208 |
|  | Diabetes | 1.273 | 0.791-2.048 | 0.319 |
| **Getting up in the morning** | Body mass index | 1.564 | 0.588-4.160 | 0.37 |
|  | Depression | 0.764 | 0.495-1.178 | 0.223 |
|  | Hypertension | 1.06 | 0.702-1.611 | 0.770 |
|  | Diabetes | 1.153 | 0.301-1.122 | 0.686 |
| **Non-snoring** | Hypertension | 0.748 | 0.244-2.291 | 0.611 |
|  | Diabetes | 0.138 | 0.018-1.08e+00 | 0.059 |
|  | Depression | 1.08 | 0.503-2.312 | 0.846 |
|  | Body mass index | 0.370 | 0.135-1.02e+00 | 0.055 |
| Depression | **ESRD** | 0.918 | 0.599-1.407 | 0.696 |
| Hypertension |  | 0.952 | 0.779-1.164 | 0.633 |
| Diabetes |  | 1.174 | 1.022-1.349 | 0.023* |
| Body mass index |  | 1.933 | 1.440-2.594 | 1.135e-05* |

CI indicates confidence interval; OR, odds ratio.

**Supplementary Table 11.** Associations of genetically predicted liability to sleep traits with ESRD after adjusting for risk factors.

| **Exposure/ Outcome** | **Adjusting** | **Multivariable MR** | | | |
| --- | --- | --- | --- | --- | --- |
|  |  | OR | 95%CI | P | FRD |
| **Insomnia/ESRD** | Depression | 3.005 | 0.435-20.773 | 0.265 | 0.265 |
| **Get up in the morning/ESRD** |  | 0.246 | 0.093-0.647 | 0.0045* | 0.0114* |
| **Non-snoring/ESRD** |  | 0.034 | 0.0028-0.407 | 0.0076* | 0.0114* |
| **Insomnia/ESRD** | Hypertension | 5.181 | 0.981-27.369 | 0.053 | 0.062 |
| **Get up in the morning/ESRD** |  | 0.334 | 0.105-1.058 | 0.062 | 0.062 |
| **Non-snoring/ESRD** |  | 0.038 | 0.0029-0.512 | 0.0135* | 0.0405* |
| **Insomnia/ESRD** | Body mass index | 0.762 | 0.175-3.321 | 0.717 | 0.717 |
| **Get up in the morning/ESRD** |  | 1.351 | 0.341-5.357 | 0.669 | 0.717 |
| **Non-snoring/ESRD** |  | 2.818 | 0.237-33.517 | 0.412 | 0.717 |
| **Insomnia/ESRD** | Diabetes | 2.736 | 0.361-20.755 | 0.33 | 0.33 |
| **Get up in the morning/ESRD** |  | 0.283 | 0.073-1.094 | 0.067 | 0.201 |
| **Non-snoring/ESRD** |  | 0.152 | 0.007-3.094 | 0.22 | 0.33 |

CI indicates confidence interval; OR, odds ratio.

**Supplementary Table 12.** STROBE-MR checklist of recommended items to address in reports of mendelian randomisation studies.

| **Item No.** | **Section** | **Checklist item** |
| --- | --- | --- |
| 1 | **Title and abstract** | Title: Sleep traits and risk of end-stage renal disease: a mendelian randomization study  Abstract:  Background: Epidemiological evidence relating sleep disorders to end-stage renal disease (ESRD) has been obscure. The present study is sought to examine sleep characteristics and ESRD.  Methods: For this analysis, we selected genetic instruments for sleep traits from published genome-wide association studies (GWAS). As instrumental variables, independent genetic variations linked with seven sleep-related features (sleep duration, getting up in the morning, daytime napping, chronotype of morning/evening person, sleeplessness /insomnia, non-snoring, and daytime dozing) were chosen. A two-sample Mendelian randomization (TSMR) study was conducted to assess the causal relationship between sleep traits and ESRD (N = 33061). The reverse MR analysis subsequently determined the causal relationship between ESRD and sleep traits. The causal effects were estimated using inverse variance weighted, MR-Egger, weighted median. To conduct sensitivity studies, Cochran's Q test, MR-Egger intercept test, MR-PRESSO, leave-one-out analysis, and funnel plot were used. To study the potential mediators, multivariable mendelian randomization analyses were undertaken further.  Results: Genetically predicted sleeplessness/ insomnia (OR = 6.11, 95%CI 1.00-37.3, P = 0.049, FDR =0.105), getting up in the morning easily(OR = 0.23, 95%CI 0.063-0.85; P = 0.0278, FDR = 0.105), non-snoring (OR = 4.76E-02, 95%CI 2.29E-03-0.985, P = 0.0488, FDR = 0.105) was suggestively associated with the risk of ESRD. However, we found no evidence favoring a causal association between other sleep traits and ESRD through the IVW method.  Conclusion: The present TSMR found no strong evidence of a bidirectional causal association between genetically predicted sleep traits and ESRD. |
|  | **Introduction** |  |
| 2 | Background | End-stage renal disease (ESRD) is a global health issue. Significant roles are played by diabetes, hypertension, and obesity in the development of ESRD. However, the etiology of ESRD remains obscure.  The exposures in this study are sleep traits, as the prevalence of sleep problems in patients with chronic kidney disease (CKD) is significant, and 44% of patients with ESRD experience sleep disturbance. A cohort study identified poor sleep quality as a predictor of ESRD and found that both short (5 hours) and long (8 hours) sleep duration were linked to the risk of ESRD. However, researchers were unable to determine if sleep disruption raises the risk of ESRD.  Mendelian randomization (MR) analysis uses genetic variants as instrumental variables (IVs) for exposure, which reduces measurement error and bias. MR is used to test exposure-outcome causal inferences. |
| 3 | Objectives | We conducted a TSMR research to examine the causative influence of seven sleep traits on ESRD. |
|  | **Methods** |  |
| 4 | Study design and data sources | Figure 1 shows the overview of the study design, and all data sources are shown in Supplementary Table 1. We conducted a bidirectional MR analysis to assess sleep traits' association with ESRD. Setting dialysis and glomerular filtration rate as additional outcomes to supplement results, and then we conducted multivariable MR to test the true causal association between sleep traits and ESRD. |
|  | a) | Setting: All data of exposures were obtained from the European group. The data of ESRD was from mixed race who have European ancestry-of-origin loci on genome-wide admixture mapping of CKD. Other outcomes are from European ancestry individuals. |
|  | b) | Participants: Summary statistics data for sleep traits from a study of UK Biobank including 337000 unrelated individuals’ self-reports. Data for ESRD from a study comprising 33061 individuals (602 cases and 32459 controls). Summary statistics for glomerular filtration rate (GFR) from a study including 32834 individuals. The outcome data for dialysis were obtained from the 5th release of the FinnGen study with 648 cases and 212841 controls. |
|  | c) | Selection of genetic variants: To determine the optimal IVs for sleep, we first extracted SNPs from published data strongly associated with sleep traits (*p*<5 × 10^-8^). Linkage disequilibrium (LD) SNPs were eliminated (r2 0.001, clumping window = 10000kb) to ensure exposure instrument independence. Then, we extracted the sleep trait instrumental factors from the ESRD GWAS and eliminated palindromic SNPs. After harmonizing exposure and outcome data, we discovered that the same allele affects both exposure and outcome. Last, we filtered SNPs with *F*-statistics greater than 10 to ensure instrument reliability and eliminate bias. In the reverse MR analysis, a more relaxed threshold was used (*p*<5 × 10^-6^) to select more SNPs of ESRD, which had been previously used in MR research. |
|  | d) | Methods of assessment: Insomnia was characterized as difficulty falling asleep at night or waking up in the middle of the night. Getting up in the morning was evaluated based on how easy it was to locate, and the significant self-report was fairly easy. The genetic connection to non-snoring was based on snoring complaints from a partner, relative, or friend. The genetic relationship of sleep duration was determined by asking people how many hours they slept per day, and the units of measurement were hours per day. Chronotype is the natural propensity for the individual to sleep at a particular time, and the morning chronotype is someone who self-reported as more of a ‘morning’ person than an 'evening' person. The assessment of GFR was based on cystatin C, and GFRcys was estimated as 76.7 × (serum cystatin C)^−1.19^. ESRD was defined as an eGFR (by the CKD-Epi Equation) of <=15 ml/min/1.73m^2^. |
|  | e) | Since this study was based on published data, no ethical approval nor informed consent was required. |
| 5 | Assumptions | Genetic instruments were associated with the risk factor of interes, were independent of potential confounders, and could only affect the outcome through the risk factor and not through alternative pathways. |
| 6 | Statistical methods: main analysis |  |
|  | a) | The genetic relationship of sleep duration was determined by asking people how many hours they slept per day, and the units of measurement were hours per day. |
|  | b) | / |
|  | c) | ESRD was modeled as a binary outcome, and models were adjusted for age, sex, race/ethnicity, study, and study center, etc. |
|  | d) | / |
|  | e) | False discovery rate (FDR) adjusted p-values proposed by Benjamini and Hochberg were used to address multiple correction testing. An FDR lower than 0.05 indicated statistical significance and supported strong evidence of a causal relationship. Associations with p<0.05 but FDR>0.05 were regarded as suggestive evidence of association. |
| 7 | Assessment of assumptions | We filtered SNPs with *F*-statistics greater than 10 to ensure instrument reliability and eliminate bias.  We conducted multivariable MR using genetic variants associated with numerous, potentially connected exposures to estimate the effect of each exposure on a single outcome. We included some risk factors as potential confounders in the sleep traits and ESRD relationship. The multivariable MR was applied to test whether there was a true causality between sleep traits and ESRD. |
| 8 | Sensitivity analyses and additional analyses | Cochran's Q test, the MR-Egger regression test and the Mendelian Randomization Pleiotropy Residual Sum and Outlier (MR-PRESSO) test were used to identify heterogeneity or pleiotropy. To check reproducibility, we ran a sensitivity analysis utilizing the leave-one-out technique. |
| 9 | Software and pre-registration |  |
|  | a) | All statistical analyses were performed using the two-sample MR package (version 0.5.6) and MR-PRESSO package (version 1.0) in R (version 4.2.1). |
|  | b) | Sleep-related characteristics and outcome data sources have been gathered and made accessible online. |
|  | **Results** |  |
| 10 | Descriptive data |  |
|  | a) | Summary statistics from IEU open GWAS project were used as the data for sleep traits, which included 337000 unrelated individuals from a study of UK Biobank. |
|  | b) | The distribution of sleep traits from UK Biobank sample are described in Supplementary Table, Additional File 1. |
|  | c) | / |
|  | d) | Participants in the ESRD study where we obtained outcome data were Hispanic/Latino, while the exposure dataset we used was all European. A study showing genome-wide admixture mapping of CKD identified European ancestry-of-origin loci in Hispanic and Latino individuals, and the locus with European ancestry was associated with the CKD risk. |
| 11 | Main results |  |
|  | a) | 28 SNPs for sleeplessness/insomnia, 37 for getting up in the morning, 22 for non-snoring, 44 for sleep duration, 80 for morning chronotype, 48 for napping during the day, and 18 for daytime dozing were extracted for MR analysis. To examine the impact of ESRD on the risk of sleep traits, we finally integrated 23 SNPs for ESRD. The additional file (see Supplementary Table 2-9, Additional File 1) provided an overview of GWAS datasets and related SNPs. |
|  | b) | According to IVW analysis, there were suggestive associations between sleeplessness/ insomnia (OR = 6.11, 95%CI 1.00-37.3, P = 0.049, FDR = 0.105, Power = 0.95), Getting up easily in the morning (OR=0.23, 95%CI 0.063-0.85; P = 0.0278, FDR = 0.105, Power = 0.1)(Figure 4), non-snoring (OR 4.759E-02, 95%CI 2.29E-02 - 0.985, P = 0.0488, FDR = 0.105, Power = 0.12) and the risk of ESRD (Table 1). For other sleep behaviors, we found no evidence of associations between genetically-predicted sleep duration (OR = 0.622, 95%CI 0.158-2.447, P = 0.49, FDR = 0.686) (See Supplementary Figure 1, Additional File 1), the morning chronotype (OR = 0.88, 95%CI 0.44-1.753, P =0.73, FDR = 0.851) (See Supplementary Figure 2, Additional File 1) and ESRD. Similarly, daytime dozing (0R = 1.2, 95%CI 4.48E-02 – 3.21E+02, P = 0.91, FDR = 0.91) (See Supplementary Figure 6, Additional File 1)and rarely daytime napping (OR = 4.15, 95%CI 0.93-18.44, P = 0.06, FDR = 0.105) (See Supplementary Figure 3, Additional File 1) was not observed evidence of having a causal association with ESRD risk. |
|  | c) | The present TSMR found no strong evidence of a bidirectional causal association between genetically predicted sleep traits and ESRD. |
|  | d) | Genetic liability to Non-snoring, getting up early, and insomnia was suggestively associated with ESRD in the primary analysis (Figure 2, 3). |
| 12 | Assessment of assumptions |  |
|  | a) | The total F value for sleep duration, getting up in the morning, chronotype, napping during the day, insomnia, non-snoring, and daytime dozing were 660.15, 547.39, 1321.85, 935.98, 423.11, 334.99, and 315.36, respectively. To examine the impact of ESRD on the risk of sleep traits, we extracted 23 SNP for ESRD with a total F-statistic of 124.05 and then removed the SNPs with F<10 to satisfy the first MR assumption. Finally, we integrated 7 SNPs for ESRD.  In the multivariable MR adjusting for diabetes, there was no evidence for a causal association of genetic liability to sleeplessness/ insomnia, getting up early in the morning and non-snoring with ESRD. Likewise, after adjustment for BMI, associations between genetic liability to sleeplessness/ insomnia, getting up early in the morning, and non-snoring and ESRD did not persist. |
|  | b) | The Cochrane Q test revealed no heterogeneity. |
| 13 | Sensitivity analyses and additional analyses |  |
|  | a) | In the sensitivity analysis, we conducted funnel pot, Cochran’s Q test, leave-one-out analysis, and MR-Egger intercept tests. The MR-Egger regression test for the sensitivity analysis revealed no horizontal pleiotropy. |
|  | b) | / |
|  | c) | In assessing the influence of ESRD on the risk of sleep traits, the IVW technique revealed no evidence favoring a causal effect of ESRD on the risk of insomnia (OR = 0.997, 95%CI = 0.990-1.003, P = 0.324), getting up early (OR =1.002, 95%CI 0.995-1.008, P =0.616), non-snoring (OR = 1.002, 95%CI 0.995-1.010, P = 0.543), sleep duration (b = -0.001, 95%CI -0.008-0.006, P = 0.783), morning chronotype (OR = 0.994, 95%CI 0.986-1.003 P = 0.173), daytime napping (OR = 0.995, 95%CI 0.989-1.001 P = 0.088) or daytime dozing (OR = 1.001, 95%CI 0.996-1.006, P = 0.598). |
|  | d) | / |
|  | e) | The leave-one-out analyses demonstrated the results' consistency. |
|  | **Discussion** |  |
| 14 | Key results | In this bidirectional TSMR investigation, sleeplessness/insomnia, waking up early, and not snoring were suggestively associated with the risk of ESRD. Furthermore, However, our data did not show evidence providing a causal connection between genetic predisposition to ESRD and sleep disturbances. Some of the associations remained after adjustment for depression and hypertension. Diabetes and BMI might partly mediate the link between sleep traits and ESRD. |
| 15 | Limitations | Our study has several strengths. This is the first MR analysis of sleep attributes with ESRD, probing evidence of the causal association between sleep-related characteristics and ESRD and studying the bidirectional causation relationship. Second, the MR design reduced the likelihood that confounding and other biases caused the observed bias. A large sample size and GWAS SNPs offered statistical validity for assessing causality. These steps improve conclusion validity.  Our study does, however, have certain shortcomings. First, participants in the ESRD where we obtained outcome data were Hispanic/Latino, while the exposure dataset we used was all European. A study showing genome-wide admixture mapping of CKD identified European ancestry-of-origin loci in Hispanic and Latino individuals, and the locus with European ancestry was associated with the CKD risk[53]. However, population stratification may contribute to confounders. The ancestry distribution, on the other hand, restricted the generalizability of our findings to other groups. Second, we could not determine if there were dose-response connections between sleep traits and ESRD. Still, uncertainty remains around the potential effects of public policy interventions on different sleep behaviors. Third, the findings of the Power analysis for non-snoring and getting up in the morning are minor, which might be due to the limited number of cases and sample size of ESRD. Finally, the results of the weighted median technique were not consistent with the suggestive associations provided by the IVW method in the primary MR analysis, indicating the presence of pleiotropy. Although we conducted sensitivity analyses that revealed no obvious pleiotropy, we find it difficult to verify the assumption that genetic instruments could only affect the outcome through the risk factor and not through pleiotropy. |
| 16 | Interpretation |  |
|  | a) | This implied that sleep disturbance might affect ESRD through depression. The causal association between insomnia and ESRD attenuated after adjusting for depression, but the associations of non-snoring and getting up early in the morning with ESRD were even more robust in multivariable MR adjusting for depression, indicating that genetic correlations between these two sleep traits and depression are less likely to be a source of biassing these findings.  Our multivariable analysis linked sleep traits to obesity, hypertension, and an increased risk of diabetes. These factors may mediate the association between sleep traits and ESRD. After adjusting for these factors, most associations between sleep traits and ESRD did not persist, indicating that these factors might confound the observed associations between sleep traits and ESRD. |
|  | b) | The precise pathophysiological mechanisms behind the link between sleep traits and ESRD remain poorly known. In this and previous research, sleep characteristics were connected to obesity, hypertension, and diabetes, and ESRD may be triggered by obesity, high blood pressure, and diabetes.  Sleep disorders were associated with a condition of the hypothalamic-pituitary-adrenal axis; snoring associated with OSA was linked to impaired autonomic nervous function. The higher risk of ESRD in patients with insomnia may be partially explained by sleep-induced alterations in the autonomic nervous system and hypothalamic-pituitary axis. Additionally, inflammation might change due to insomnia, primary snoring, and obstructive sleep apnea. At the same time, systemic and local chronic inflammation (in the kidney) operate as risk factors for diabetic renal disease and its development into ESRD. |
|  | c) | Still, uncertainty remains around the potential effects of public policy interventions on different sleep behaviors. |
| 17 | Generalisability | However, population stratification may contribute to confounders. The ancestry distribution, on the other hand, restricted the generalizability of our findings to other groups. |
|  | **Other information** |  |
| 18 | Funding | This study was supported by the Research Fund of Early Biological Markers of Geriatric Hyperalgesia and Early Identification Protocols for Multimorbidity Co-Morbidities in The Population (2020YFC2005002). |
| 19 | Data and data sharing | GWAS summary statistics for sleep traits are publicly available through http://www.nealelab.is/blog/2017/7/19/rapid-gwas-of-thousands-of-phenotypes-for-337000-samples-in-the-uk-biobank. The summary statistics of GWAS for ESRD are derived from a GWAS conducted by Wojcik GL et al.(https://doi.org/10.1038/s41586-019-1310-4). The data for dialysis is derived from https://www.finngen.fi/en. The summary data for the glomerular filtration rate is from a GWAS conducted by Pattaro C et al.( https://doi.org/10.1038/ncomms10023). In the assessment of risk factors, summary statistics for depression and hypertension are avalaible through https://www.finngen.fi/en. Summary statistics for body mass index (BMI) can be download from https://doi.org/10.7554/eLife.34408. The summary statistics of GWAS for diabetes are derived from a GWAS conducted by Xue A et al.( https://doi.org/10.1038/s41467-018-04951-w). All data can be downloaded from IEU OpenGWAS project (https://doi.org/10.1101/2020.08.10.244293). |
| 20 | Conflicts of interest | All authors declare no conflict of interest. |

**Supplementary Figure 1. Forest plot (1), sensitivity analysis (2), scatter plot (3) and funnel plot (4) of the causal effect of Sleep duration on ESRD risk.**


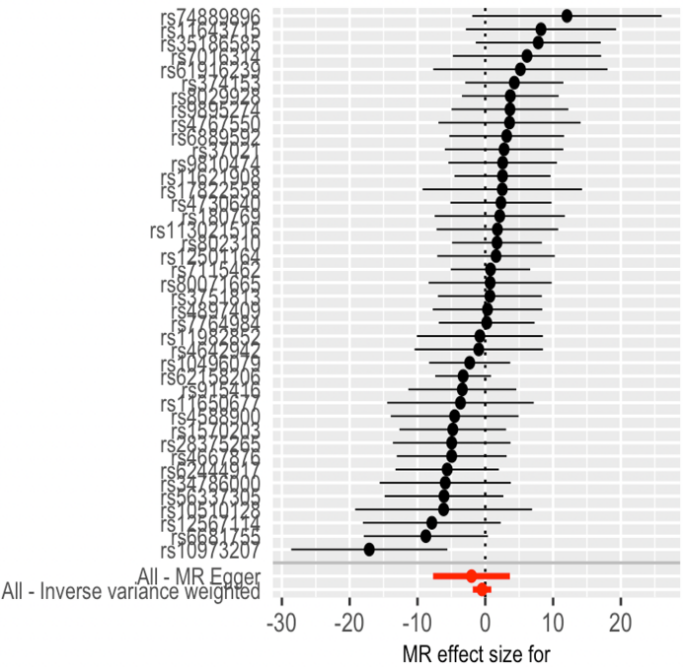

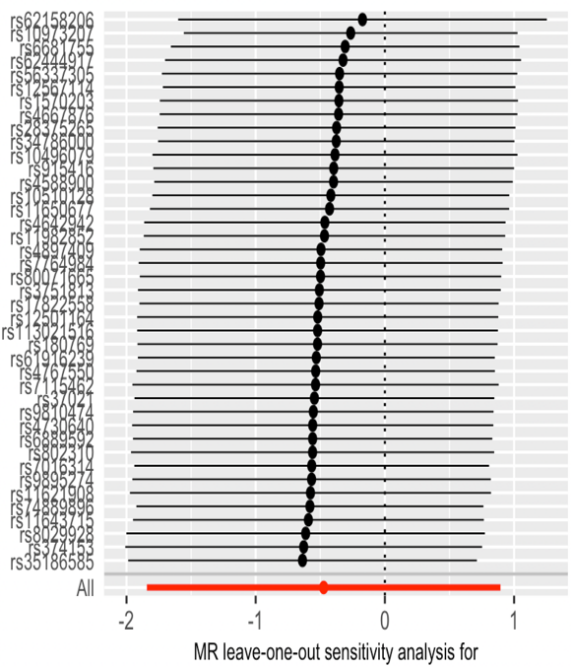


**A B**

**
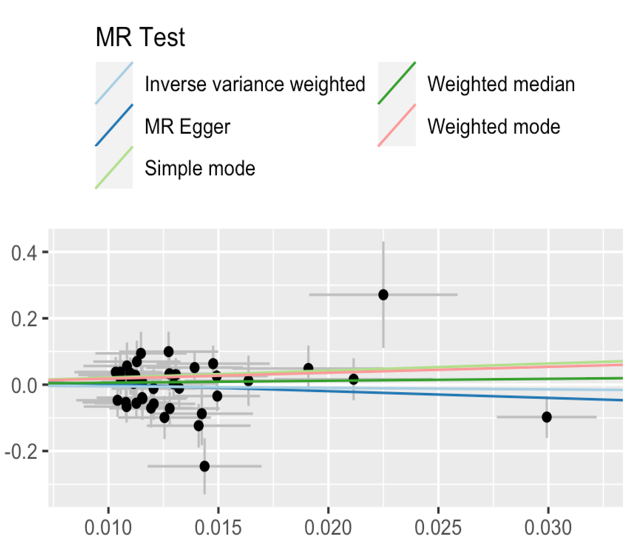

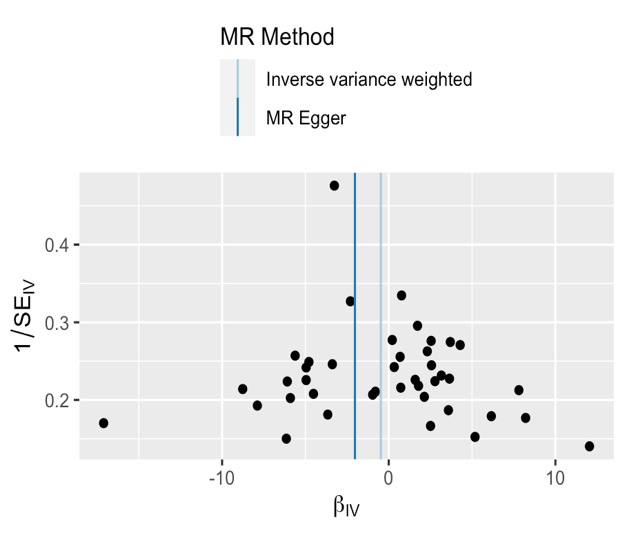
**

**C D**

**Supplementary Figure 2. Forest plot (1), sensitivity analysis (2), scatter plot (3) and funnel plot (4) of the causal effect of chronotype on ESRD risk.**

**
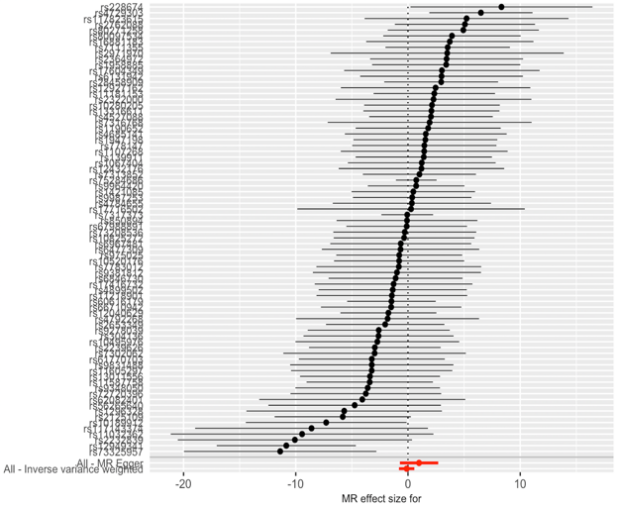

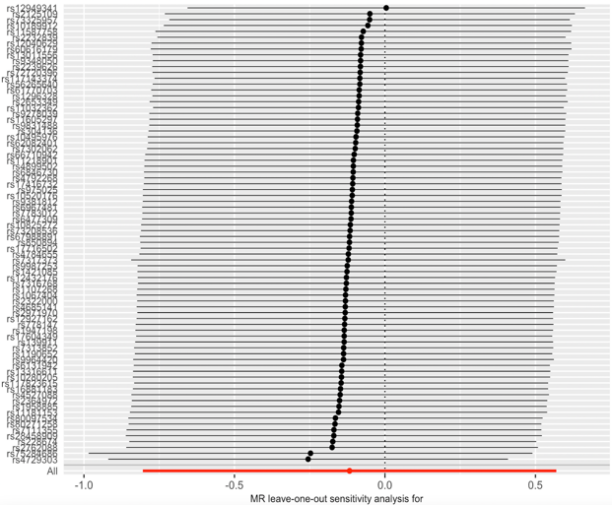
**

**A B**

**
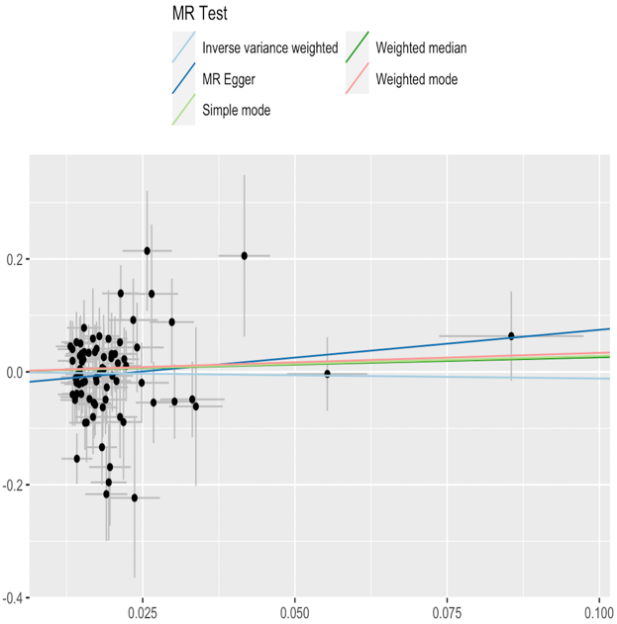

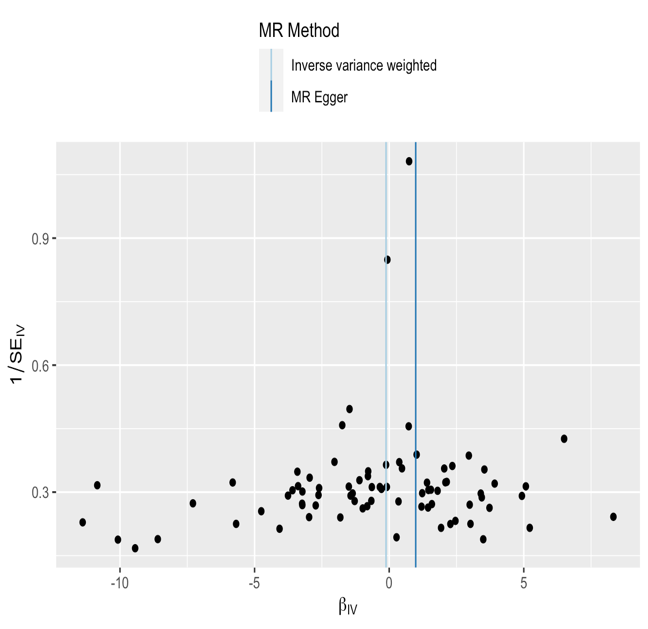
**

**C D**

**Supplementary Figure 3. Forest plot (1), sensitivity analysis (2), scatter plot (3) and funnel plot (4) of the causal effect of Napping during the day on ESRD risk.**

**
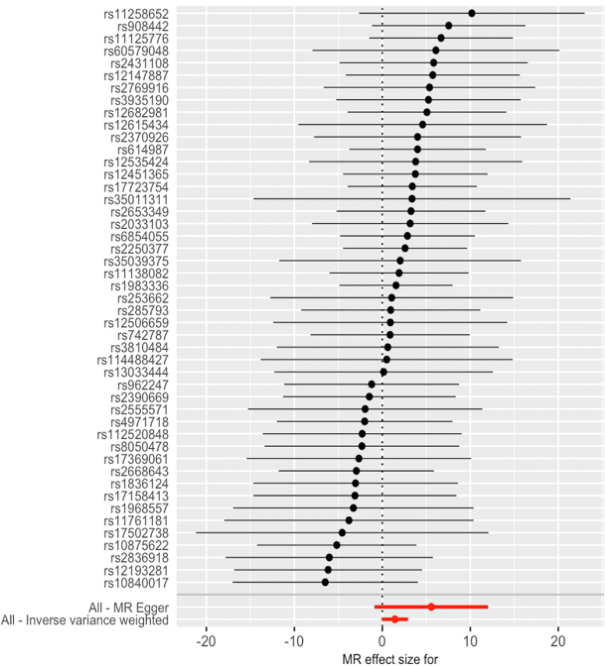

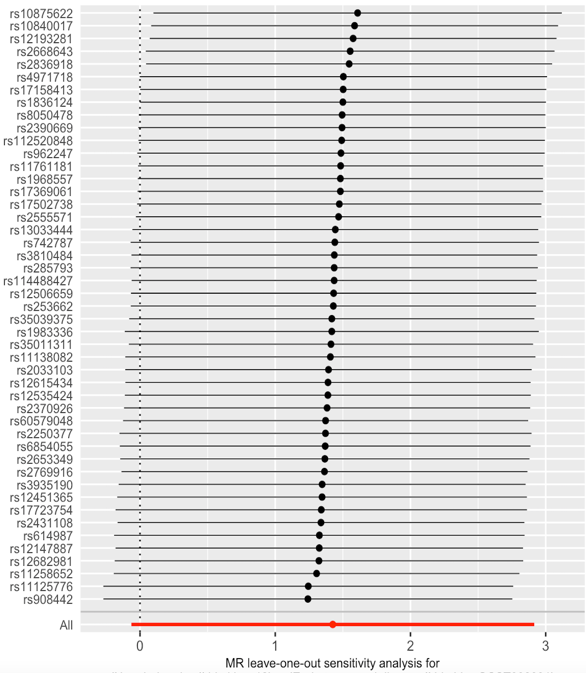
**

**A B**

**
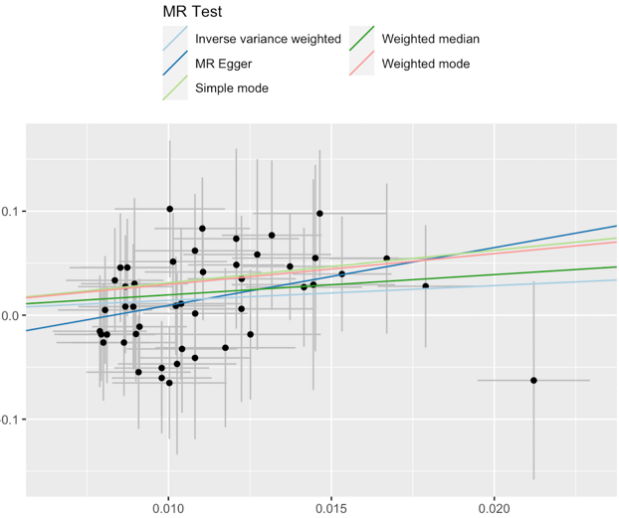

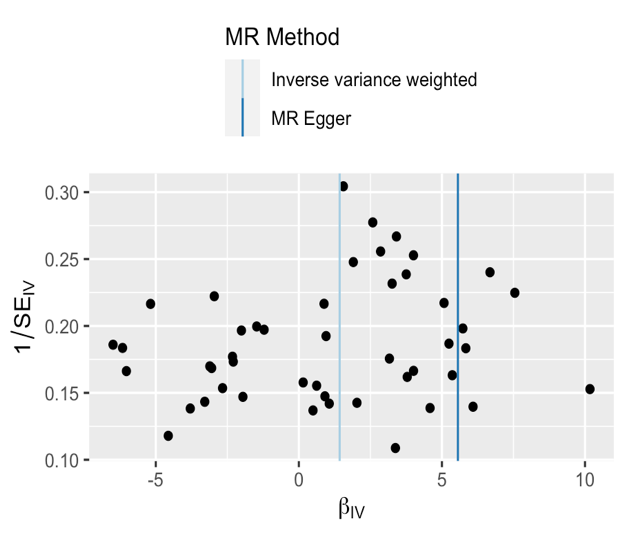
**

**C D**

**Supplementary Figure 4. Forest plot (1), sensitivity analysis (2), scatter plot (3) and funnel plot (4) of the causal effect of Insomnia on ESRD risk.**

**
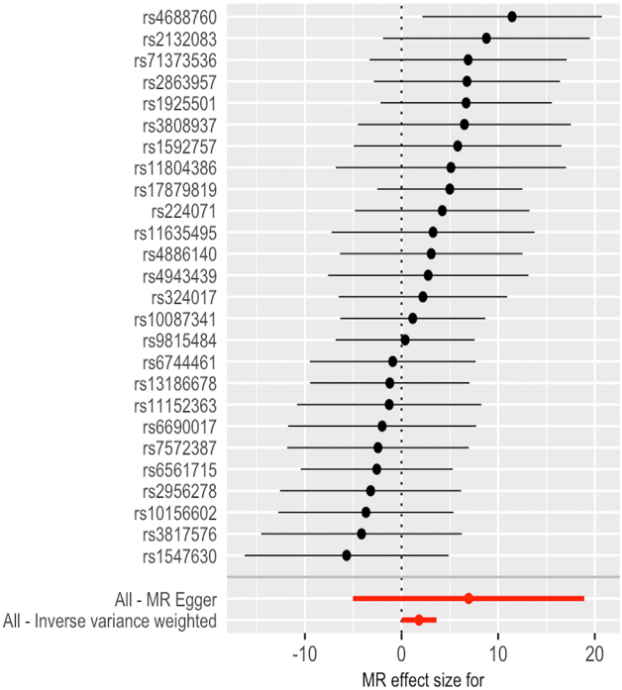

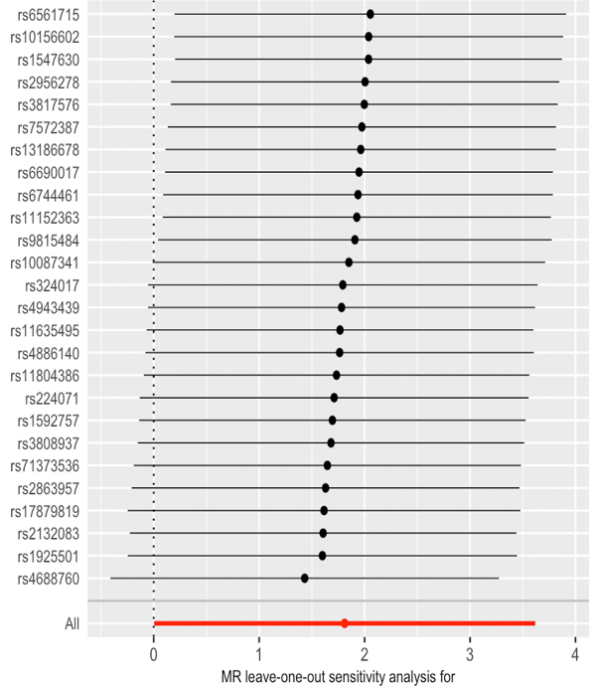
**

**A B**

**
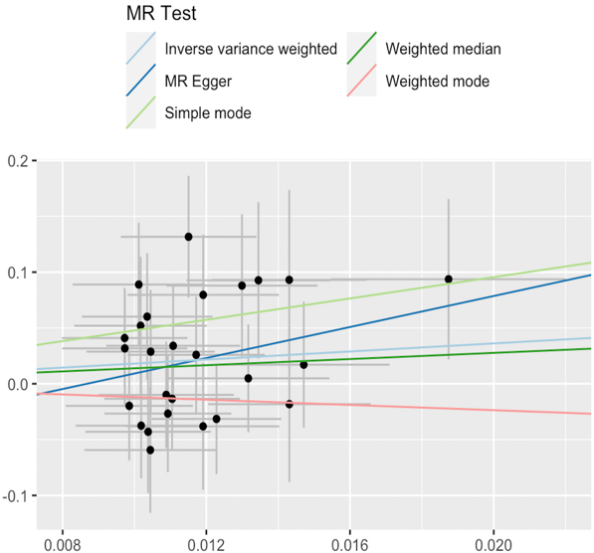

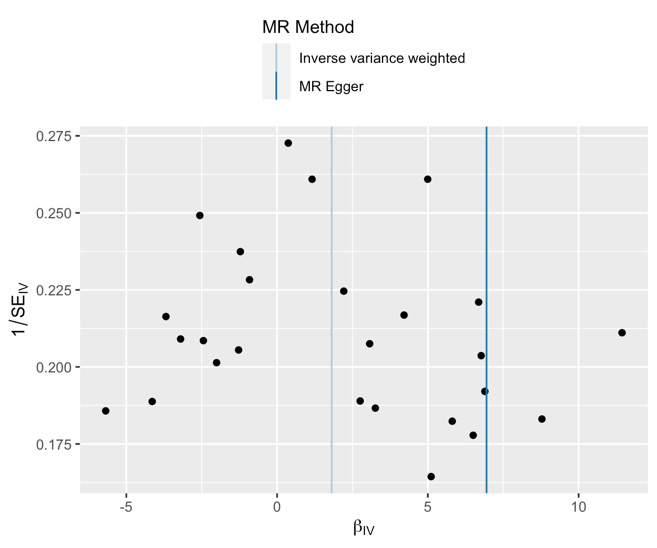
**

**C D**

**Supplementary Figure 5. Forest plot (1), sensitivity analysis (2), scatter plot (3) and funnel plot (4) of the causal effect of Non-snoring on ESRD risk.**

**
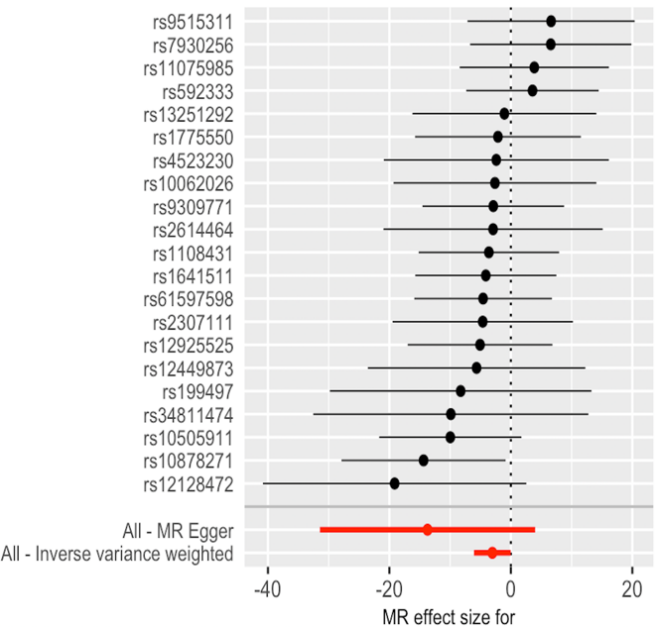

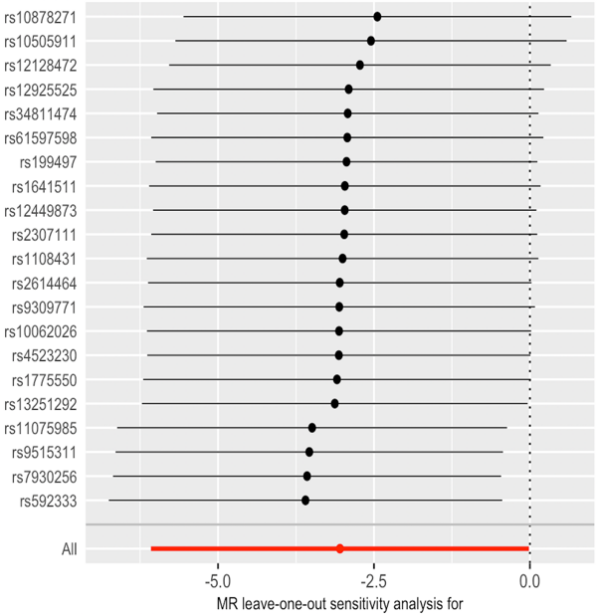
**

**A B**

**
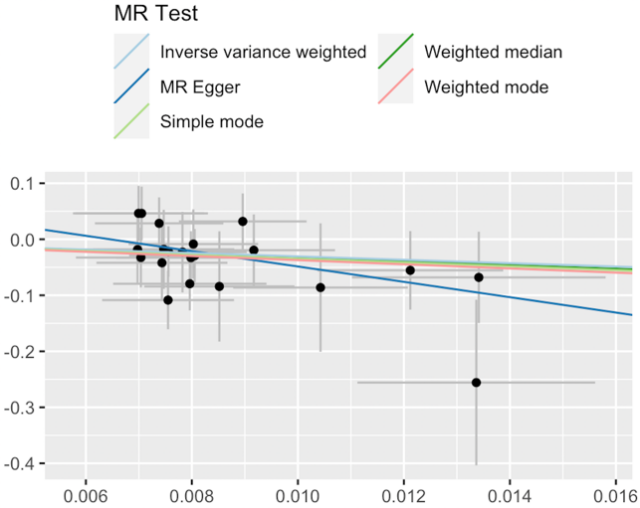

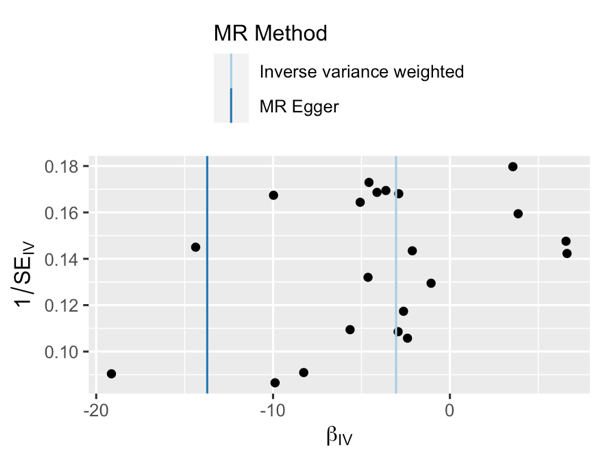
**

**C D**

**Supplementary Figure 6. Forest plot (1), sensitivity analysis (2), scatter plot (3) and funnel plot (4) of the causal effect of daytime dozing on ESRD risk.**

**
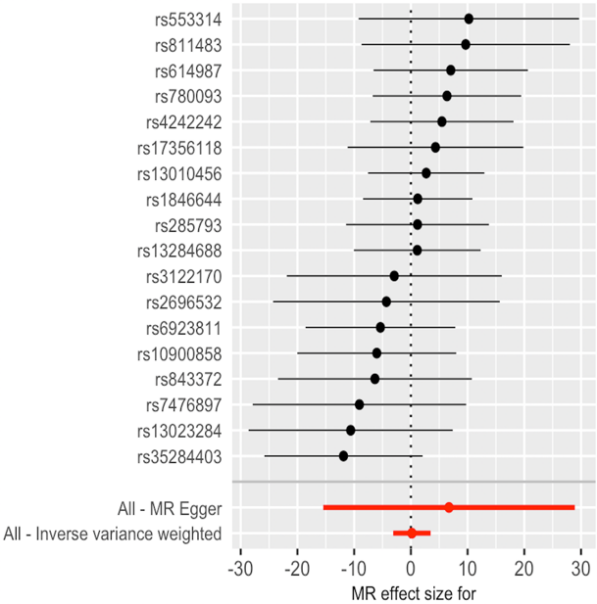

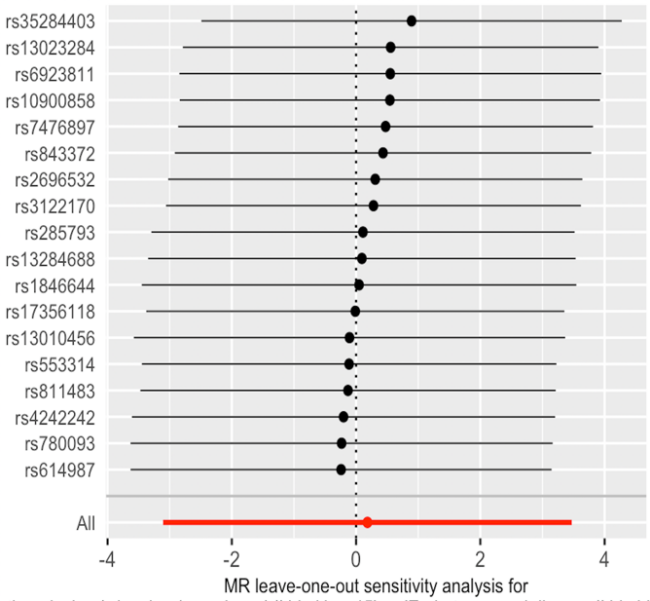
**

**A B**

**
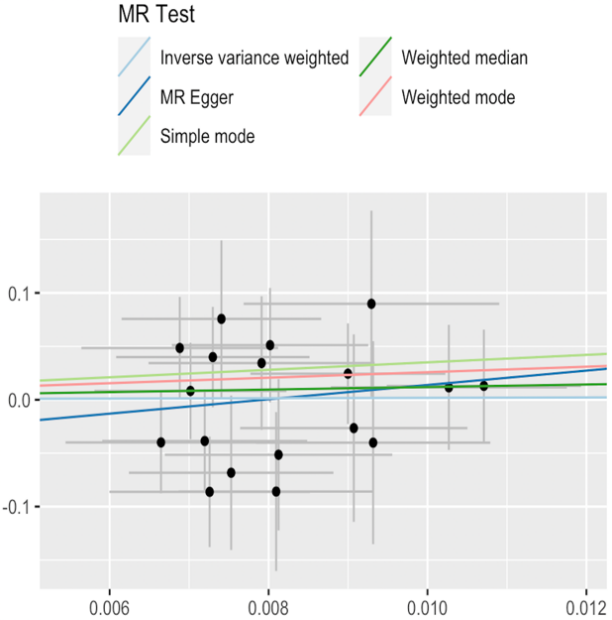

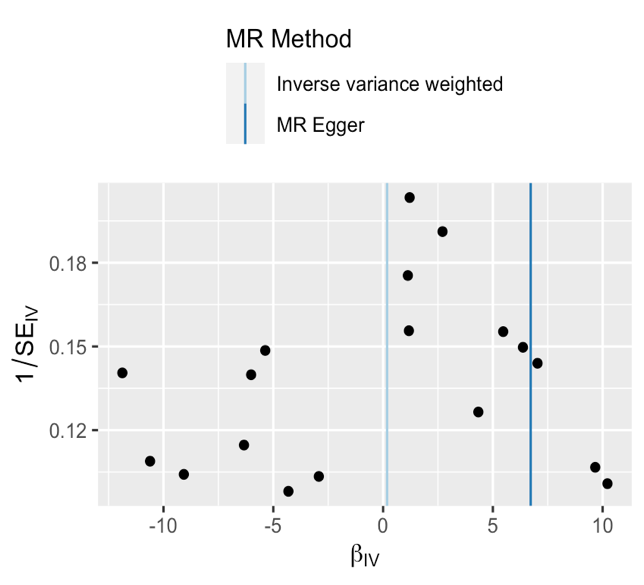
**

**C D**
